# Large-Scale Gene Disruption in *Magnaporthe oryzae* Identifies MC69, a Secreted Protein Required for Infection by Monocot and Dicot Fungal Pathogens

**DOI:** 10.1371/journal.ppat.1002711

**Published:** 2012-05-10

**Authors:** Hiromasa Saitoh, Shizuko Fujisawa, Chikako Mitsuoka, Akiko Ito, Akiko Hirabuchi, Kyoko Ikeda, Hiroki Irieda, Kae Yoshino, Kentaro Yoshida, Hideo Matsumura, Yukio Tosa, Joe Win, Sophien Kamoun, Yoshitaka Takano, Ryohei Terauchi

**Affiliations:** 1 Iwate Biotechnology Research Center, Kitakami, Iwate, Japan; 2 Laboratory of Plant Pathology, Graduate School of Agriculture, Kyoto University, Kyoto, Japan; 3 The Sainsbury Laboratory, John Innes Centre, Norwich, United Kingdom; 4 Laboratory of Plant Pathology, Graduate School of Agricultural Sciences, Kobe University, Kobe, Japan; Oregon State University, United States of America

## Abstract

To search for virulence effector genes of the rice blast fungus, *Magnaporthe oryzae*, we carried out a large-scale targeted disruption of genes for 78 putative secreted proteins that are expressed during the early stages of infection of *M. oryzae*. Disruption of the majority of genes did not affect growth, conidiation, or pathogenicity of *M. oryzae*. One exception was the gene *MC69*. The *mc69* mutant showed a severe reduction in blast symptoms on rice and barley, indicating the importance of MC69 for pathogenicity of *M. oryzae*. The *mc69* mutant did not exhibit changes in saprophytic growth and conidiation. Microscopic analysis of infection behavior in the *mc69* mutant revealed that MC69 is dispensable for appressorium formation. However, *mc69* mutant failed to develop invasive hyphae after appressorium formation in rice leaf sheath, indicating a critical role of MC69 in interaction with host plants. *MC69* encodes a hypothetical 54 amino acids protein with a signal peptide. Live-cell imaging suggested that fluorescently labeled MC69 was not translocated into rice cytoplasm. Site-directed mutagenesis of two conserved cysteine residues (Cys36 and Cys46) in the mature MC69 impaired function of MC69 without affecting its secretion, suggesting the importance of the disulfide bond in MC69 pathogenicity function. Furthermore, deletion of the *MC69* orthologous gene reduced pathogenicity of the cucumber anthracnose fungus *Colletotrichum orbiculare* on both cucumber and *Nicotiana benthamiana* leaves. We conclude that MC69 is a secreted pathogenicity protein commonly required for infection of two different plant pathogenic fungi, *M. oryzae* and *C. orbiculare* pathogenic on monocot and dicot plants, respectively.

## Author Summary

*Magnaporthe oryzae* causes the most devastating fungal disease in rice. *M. oryzae* secretes a plethora of effector proteins, including several avirulence proteins which are known to be recognized by host resistance proteins activating innate immunity. However, the effectors that are required for virulence activity have not been identified in *M. oryzae* to date except for an effector protein, Secreted LysM Protein 1 (Slp1) that was recently identified. We performed a large-scale disruption analysis of *M. oryzae* effector candidates and identified a small protein MC69, which is secreted by the fungus during infection. When MC69 is absent, pathogenicity is severely reduced after penetration into the host cells. Furthermore, deletion of the *MC69* orthologous gene in *Colletotrichum orbiculare* reduced its pathogenicity in the host plants cucumber and *Nicotiana benthamiana*. Thus, MC69 is conserved in ascomycete fungi and is crucial for establishing compatibility. This is the first report of a single secreted protein that is indispensable for pathogenicity in both monocot and dicot pathogenic fungi. How MC69 contributes to pathogenicity or virulence is unknown but it could be required for the fungus to be a pathogen or might be a classical effector that acts on plant target molecules.

## Introduction

Rice blast, caused by an ascomycete fungus *Magnaporthe oryzae*, is the most severe fungal disease of rice throughout the world [1]. Genetic studies of this pathogen over the last two decades have made the *Magnaporthe*-rice pathosystem an excellent model for investigating fungus-plant interactions.

Plants are equipped to sense evolutionarily conserved microbial molecular signatures, collectively called *P*athogen-*A*ssociated *M*olecular *P*atterns (PAMPs) or *M*icrobe-*A*ssociated *M*olecular *P*atterns (MAMPs), and activate *P*AMP-*T*riggered *I*mmunity (PTI) [2]–[4]. Pathogens are capable of inhibiting PTI on their host plants by delivering virulence effector proteins into host cells [5]–[9].

In *M. oryzae*, effector secretion machinery has recently been elucidated [10]–[12]. A Golgi-localized P-type ATPase-encoding gene, *MgAPT2* is required for exocytosis during plant infection. Further analysis suggested that *MgAPT2* is involved in secretion of a range of extracellular enzymes as well as an AVR effector for the rapid induction of host defense responses in an incompatible reaction in rice cultivar IR-68 [10]. Another study demonstrated that *M. oryzae* mutants with a defect in an ER chaperone-encoding gene, *LHS1*, have reduced activities of extracellular enzymes and secretion of AVR-Pita1 [13], [14] blocking *Pi-ta R*-gene-mediated hypersensitive response. The contribution of *LHS1* to protein translocation and secretion of proteins, including effectors, revealed the importance of ER chaperones for successful disease development by rice blast fungus [12]. Live-cell imaging revealed development of the biotrophic interfacial complex (BIC), a structure that accumulates fluorescently labeled effectors secreted by invasive hyphae (IH). The examined BIC-localized secreted proteins were translocated into rice cytoplasm. By contrast, a biotrophy-associated secreted protein BAS4, which uniformly outlines the IH, was not translocated into the host cytoplasm [11]. These results suggest that BIC represents the site of effector translocation in rice blast disease [11].

Several effector protein genes have been cloned and characterized from *M. oryzae* but all of them were avirulence (AVR) effectors with no virulence function elucidated to date [13]–[20] except for a recently identified virulence effector protein, Slp1 [21]. Slp1 accumulates at the interface between the fungal cell wall and the rice plasma membrane, can bind to chitin, and is able to suppress chitin-induced plant immune responses, including generation of reactive oxygen species and plant defense gene expression [21]. Several effector candidates were identified by using interaction transcriptome in the biotrophic invasion of *M. oryzae* [22]. In the paper the authors have identified a known effector PWL2 as well as 58 candidate effectors showing >10-fold increase in the expression in the biotrophic invasive hyphae relative to control mycelia using *M. oryzae* oligoarrays. Four of these candidates were confirmed to be fungal biotrophy-associated secreted proteins [22]. However, virulence function of all the candidates has not been elucidated, and comprehensive gene disruption analyses of the candidates have not been carried out. Therefore, in this study we employed a large-scale disruption analysis of *M. oryzae* secreted protein genes to search for novel virulence effectors.

Whole-genome draft sequence of *M. oryzae* was published for the isolate 70-15, a laboratory strain [23]. The genome assembly consists of 37.8 Mb nucleotides encoding 11,109 predicted protein coding genes. We recently retrieved 1,306 putative secreted protein genes from the predicted proteome of 70-15 [20]. From these, a total of 78 genes expressed in the fungus were disrupted and analyzed. We found that disruptants of the 77 genes did not show change in pathogenicity as compared to the wild-type strains. Disruption of only one gene, *MC69*, showed a severe reduction in pathogenicity. Further analysis showed that MC69 protein is involved in the full pathogenicity of *M. oryzae* after the penetration stage of infection.

## Results

### Large-scale disruption analysis of *Magnaporthe oryzae* secreted protein genes

To search for effector protein genes of *Magnaporthe oryzae*, we carried out a large-scale targeted gene disruption analysis of the 78 putative secreted protein genes that are expressed during infection (Table 1). Initially we selected 1,306 putative secreted protein genes as described previously [20]. The 78 genes subjected to functional study were selected on the basis of their confirmed expression in the pathogen at the early stages of infection. We focused on secreted protein genes involved in the two stages of infection of *M. oryzae*. One is the appressorium formation stage and the other is the biotrophic invasion stage. Treatment with cyclic AMP (cAMP) induces appressorium formation on hydrophilic surface [24]. By SAGE (Serial Analysis of Gene Expression) of cAMP-treated conidia on hydrophilic membrane 6 h after the start of treatment [25], we identified several pathogenicity genes, e.g. *MPG1*, *MAS1* and *MAC1*, already characterized [26]–[28] and thought to be involved in pathogen-host interaction. Therefore, we assumed that a part of effector genes should be expressed during the appressorium formation. To achieve a high efficiency tag-to-gene annotation, we established SuperSAGE method that extracts a 26-bp tag from each cDNA [29]. SuperSAGE of the cAMP-treated *M. oryzae* strain 70-15 has been done in this study to search for novel effector candidates (Table S1). Furthermore, we also used the SuperSAGE data of invasive hyphae for searching new effectors (Supplemental Data Set 1 in [20]). Indeed, this SuperSAGE analysis revealed that two *AVR* effector genes, *AVR-Pia* and *AVR-Pii* were expressed at the stage of invasive hyphae (Supplemental Data Set 2 in [20]).

**Table 1:** Gene disruption analysis of 78 putative secreted protein genes.

Mutant ID	Gene ID	cAMP^a^	IH^b^	Mutant ID	Gene ID	cAMP^a^	IH^b^
HS9	MGG_04172.6	+	−	MC28	MGG_01609.6	+	+
RT11	MGG_08342.6	+	−	MC33	MGG_09378.6	+	−
RT76	MGG_05716.6	+	−	MC45	MGG_00380.6	−	+
HM4	MGG_04757.6	+	+	MC47	MGG_00269.6	−	+
HM5	MGG_09188.6	+	−	MC48	MGG_05989.6	−	+
HM6	MGG_05798.6	−	+	MC52	MGG_10171.6	−	+
HM17	MGG_09920.6	+	−	MC55	MGG_05366.6	+	−
HM18	MGG_05785.6	+	+	MC56	MGG_05608.6	+	−
HM20	MGG_03356.6	−	+	MC57	MGG_10877.5	+	−
HM21	MGG_00505.6	+	+	MC58	MGG_08275.6	+	+
HM22	MGG_07763.6	−	+	MC59	MGG_09246.6	+	−
HM24	MGG_02245.6	−	+	MC61	MGG_09716.6	+	−
HM27	MGG_10102.6	−	+	MC62	MGG_02296.6	+	−
HM30	MGG_05381.6	+	−	MC63	MGG_06069.6	+	+
HM36	MGG_09460.6	+	−	MC65	MGG_05912.6	+	+
HM57	MGG_00703.6	+	+	MC69	MGG_02848.6	+	−
HM63	MGG_00659.6	+	−	MC70	MGG_03347.6	+	−
HM65	MGG_03245.6	+	+	MC71	MGG_12906.6	+	−
HM66	MGG_02420.6	−	+	MC72	MGG_13009.6	+	−
HM68	MGG_01532.6	+	+	MC73	MGG_13275.6	+	−
HM88	MGG_06951.6	+	+	MC79	MGG_08041.6	+	+
HM91	MGG_00314.6	+	−	MC81	MGG_09875.6	−	+
HM93	MGG_01843.6	+	+	MC82	MGG_07560.6	+	+
HM104	MGG_02987.6	+	+	MC83	MGG_00052.6	+	+
HM106	MGG_03130.6	+	+	MoCel12A	MGG_00677.6	−	+
HM108	MGG_07312.6	+	−	KY5	MGG_10799.6	+	+
Eco2	MGG_00269.6	−	+	KY8	MGG_03844.6	−	+
HMM14	MGG_01872.6	+	+	KY10	MGG_03870.6	−	+
HMM53	MGG_06216.6	+	+	KY22	MGG_10291.6	−	+
Taka1	MGG_05232.6	+	−	KY23	MGG_10394.6	−	+
Taka2	MGG_00860.6	+	−	KY45	MGG_02898.6	−	+
MC4	MGG_06840.6	−	+	KY51	MGG_01387.6	−	+
MC8	MGG_07877.6	+	+	KY55	MGG_03338.6	−	+
MC11	MGG_03593.6	+	+	AI9	MGG_09742.6	+	+
MC14	MGG_09465.6	+	+	AI41	MGG_07704.6	+	+
MC16	MGG_00552.6	+	+	AI43	MGG_05092.6	+	+
MC19	MGG_05103.6	−	+	AI44	MGG_03316.6	−	+
MC24	MGG_04952.6	−	+	AI58	MGG_07645.6	+	−
MC25	MGG_03276.6	−	+	AI59	MGG_01064.6	+	+

a Expressed gene in the cAMP-treated *M. oryzae* on dialysis membrane at 6 hpi in the Table S1.

b Expressed gene in the M. oryzae-infected rice leaf sheath at 40 hpi in the Supplemental data set 1 [20].

(+) or (−) indicate the expressed gene or not, respectively in the cAMP-treated or invasive hyphae of *M. oryzae*.

**Figure 1:**
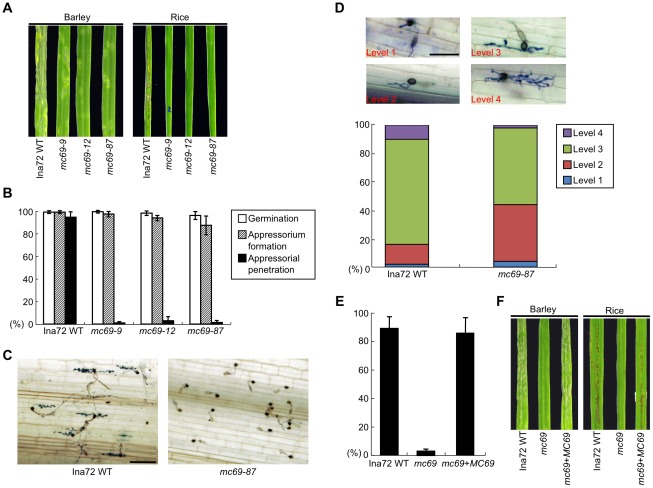
*MC69* is involved in appressorial penetration and pathogenicity of *M. oryzae*. (A) *MC69* is required for pathogenicity of *M. oryzae* strain Ina72. Conidial suspension of the wild-type strain Ina72 (Ina72 WT) and the *mc69* mutants (*mc69-9*, *-12*, *-87*) were inoculated on barley (cv. Nigrate) and rice (cv. Shin No. 2) leaves, and incubated for 4 and 7 days, respectively. (B) Germination, appressorium formation and appressorial penetration of Ina72 WT and the *mc69* mutants. The ratio of germination was calculated as the mean percentage of conidia germinated after 24 h on rice (cv. Shin No. 2) leaf sheath cells. The mean percentage of appressorium formation on rice leaf sheath cells among the germinated conidia is presented. Three replicates of ∼50 conidia were counted for each observation. The mean percentage of appressorial penetration by the *mc69* mutants 32 h after inoculation is presented. Standard errors are indicated by the vertical bars. (C) Appressorial penetration assays on rice leaf sheath cells. Conidia from Ina72 WT and *mc69-87* germinated and formed melanized appressoria. All appressoria formed by Ina72 WT penetrated and produced infectious hyphae but no appressoria formed by *mc69-87* produced infectious hyphae in most of area. Photographs were taken 32 h after inoculation. Scale bar = 40 µm. (D) Invasive growth rating of rice leaf sheath cells 32 h after inoculating with Ina72 WT and *mc69-87*. The levels for invasive growth rating are given above. For details of the invasive growth levels and rating see Materials and Methods. Scale bar = 20 µm. (E), (F) Complementation of *mc69* mutant with the wild type allele of *MC69*. (E) Appressorial penetration by Ina72 WT, *mc69-87* (*mc69*) and the *MC69* re-introduced strain (*mc69*+*MC69*). Mean percentage of appressorial penetration is recorded 32 h after inoculation in rice leaf sheath cells. Four replicates of ∼50 appressoria were counted for each observation. (F) Blast symptoms caused by Ina72 WT, *mc69* and *mc69*+*MC69* on barley (cv. Nigrate) and on rice (cv. Shin No 2) 3 and 4 days after inoculation, respectively.

To investigate the function of the effector candidate genes, we generated disruption mutants for each of the selected 78 genes (Table 1) in *M. oryzae* by TAG-KO method [30], [31]. To assess the virulence of each mutant, conidial suspension of each mutant was sprayed onto seedlings of a barley cultivar Nigrate, which is susceptible to the wild type *M. oryzae*. Blast phenotypes of barley infected by KO mutants of all the genes except for *MC69* gene were the same as that infected by wild-type strain Ina72. Similarly the 77 mutants did not show reduced virulence in a susceptible rice cultivar Shin No. 2. By contrast, we observed a dramatic reduction in disease symptoms on barley cotyledons and the susceptible rice cultivar, Shin No. 2, inoculated with all of the three independent *mc69* mutant lines (Figure 1A). Consequently, we identified the *MC69* gene (MGG_02848.6) as required for pathogenicity of *M. oryzae* after a large-scale targeted gene disruption analysis of the 78 putative secreted protein genes.

In summary, we found that targeted disruption of *MC69* affected pathogenicity of *M. oryzae* and disruption of the other 77 genes had no effect on its pathogenicity.

### *MC69* is required for appressorial penetration and pathogenicity of *M. oryzae*

To investigate the physiological and molecular function of MC69 in detail, we generated *MC69* disruptants in *M. oryzae* strain Ina72 by targeted gene disruption as described above. Colony growth, color and the production of conidia were the same as the wild-type strain (Figure S2A and B). We observed a remarkable reduction in disease symptoms on barley and rice inoculated with the *mc69* mutants compared to those inoculated with the wild type strain 4 and 7 days after inoculation suggesting an important role of *MC69* in fungal pathogenicity (Figure 1A). Subsequently, we performed a detailed phenotypic analysis of the *mc69* mutants. The *mc69* mutants exhibited a defect in appressorium-mediated penetration in rice leaf sheath cells but neither in conidial germination nor appressorium formation (Figure 1B and C). We studied 200 appressoria of each *mc69* mutant, 97∼99% with failed penetration (no visible hyphae) and 1∼2% with post-penetration blockage. Therefore, we conclude that *MC69* is required for appressorial penetration and pathogenicity of *M. oryzae*. Although most of appressoria formed by the *mc69* mutants could neither penetrate nor produce infectious hyphae in the inoculated rice leaf sheath cells, we further analyzed invasive growth at 50 appressorial penetration sites by rating the hyphal growth from the level 1 (low) to 4 (high; see Materials and Methods, Figure 1D). In Ina72 WT, 84% of penetration sites showed invasive growth levels 3 or 4, by contrast in the *mc69* mutant infectious growth within the inner epidermal tissue was relatively limited (levels 2 and 3) 32 hours after inoculation, suggesting that the loss of *MC69* also affects infectious growth to some extent at the post-penetration stage (Figure 1D). To test whether the observed phenotypes of the *mc69* mutants were solely caused by disruption of *MC69*, an intact copy of *MC69* was introduced into the *mc69* mutant *mc69-87* (Figure S1B) for complementation. The *MC69*-reintegrated strain showed normal appressorial penetration rate and the strain developed blast disease symptoms on barley and rice leaves with a similar extent to the wild type (Figure 1E and F). These results demonstrate that disruption of *MC69* gene caused defects in appressorial penetration and development of blast symptoms by *M. oryzae*.

### MC69 is expressed in conidia and all stages of infection, secreted but not translocated into rice cells

*MC69* was found in the SuperSAGE list of the cAMP-treated conidia (Table S1). *MC69*-EST was found in MGOS databases for mycelium, conidia, germinated conidia and appressoria [32]. RL-SAGE tags of *MC69* were also found in the fungus grown on a minimum medium for three days [33]. These data suggest that *MC69* is constitutively expressed in *M. oryzae*.

**Figure 2:**
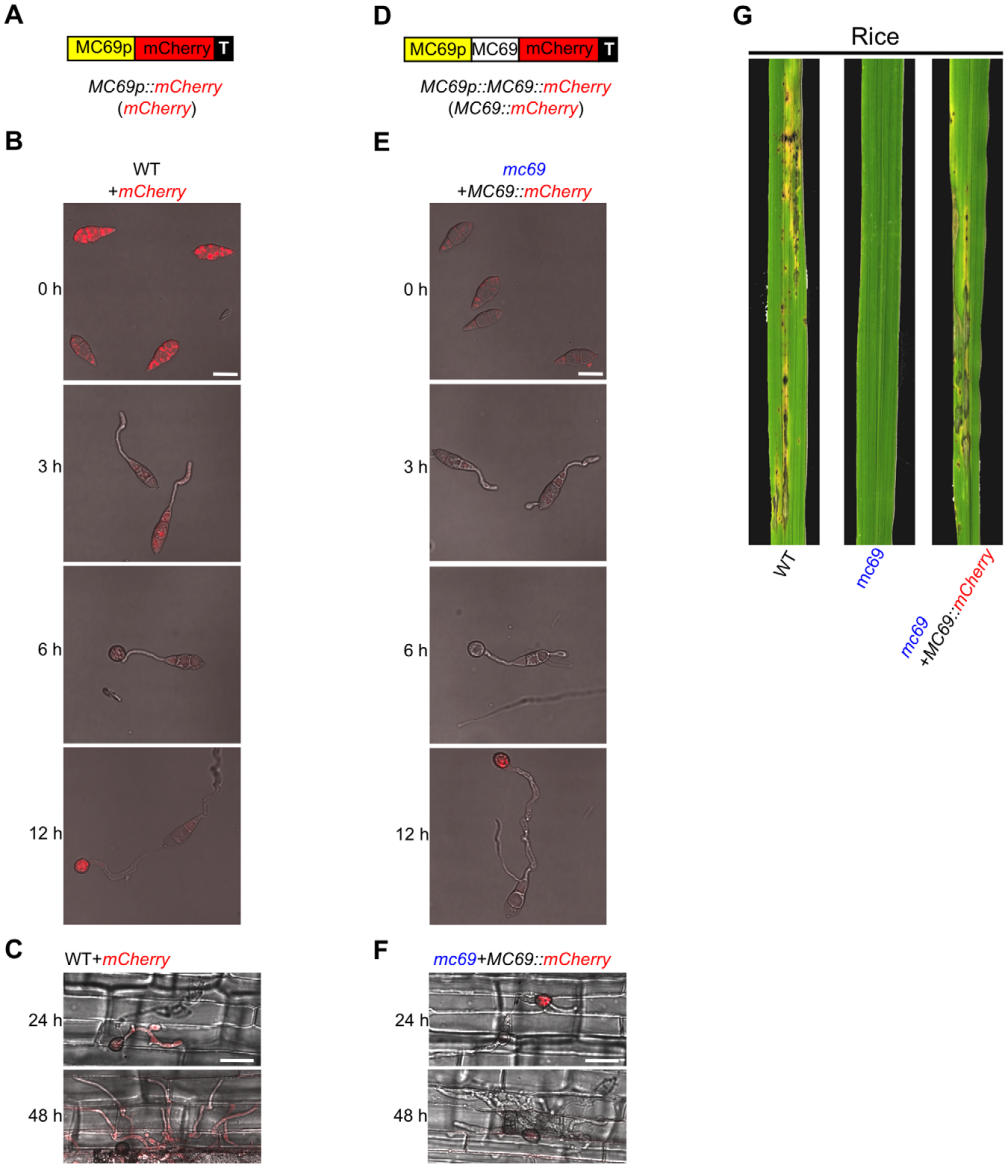
Microscopic analysis suggests that *MC69* promoter is constitutively active and MC69::mCherry fusion protein is secreted. (A) and (D) Schematic diagrams of mCherry and MC69::mCherry fusion protein expression constructs. (B) and (E) Conidia from WT+*mCherry* and *mc69*+*MC69::mCherry* were harvested, and appressorium development was observed for 12 h on glass coverslips. Merged DIC and mCherry (red) images were taken. Scale bars = 10 µm. (C) and (F) Merged DIC and mCherry images of the rice leaf sheath cells infected with mCherry- and MC69::mCherry-expressing transformants 24 h and 48 h after inoculation. Scale bars = 20 µm. (G) Blast symptoms caused by Ina72 WT, *mc69* and *mc69*+*MC69::Cherrry* on rice (cv. Shin No. 2) 7 days after inoculation.

To investigate the expression pattern of *MC69* in detail, we produced an *M. oryzae* strain Ina72 harboring a vector containing the *MC69* promoter fused with a reporter protein gene *mCherry* (*MC69p::mCherry*; Figure 2A) generating WT+*mCherry*. The mCherry fluorescence was observed in all morphological stages with enhanced fluorescence in conidia before germination (0 h) and matured appressoria (12 h after incubation) on glass coverslips under confocal laser-scanning microscope (Figure 2B). To determine the mode of expression and spatial localization of the MC69 protein, a construct *MC69p::MC69::mCherry* was prepared (Figure 2D) and used for transformation of the *mc69* mutant generating *mc69*+*MC69::mCherry*, and the mCherry fluorescence was then observed on glass coverslips (Figure 2E). The transgenic *M. oryzae* mutant *mc69* expressing *MC69::mCherry* restored pathogenicity (Figure 2G), showing that the fusion protein MC69::mCherry is functional for infectivity. The mCherry fluorescence was detected in all the developmental stages like WT+*mCherry*, but the intensity of fluorescence in the strain *mc69*+*MC69::mCherry* was weaker than that of the strain WT+*mCherry* presumably because of secretion and diffusion of MC69::mCherry fusion protein (Figure 2B and E). To observe the fluorescence of WT+*mCherry* and *mc69*+*MC69::mCherry* in the infected tissues, we inoculated these conidial suspensions to rice leaf sheath. The mCherry fluorescence was detected in the invaded hyphae 24 and 48 hours after inoculation with WT+*mCherry*, but not detected in that inoculation with *mc69*+*MC69::mCherry* (Figure 2C and F). These results suggest that the *MC69* gene is expressed throughout infection: conidia, infection-related morphogenesis and subsequent growth stage. The fluorescence from MC69::mCherry fusion proteins was not detected in the invaded hyphae *in planta* presumably because they have been secreted.

To obtain direct evidence that the MC69 protein is actually produced in the invasive hyphae, we made *mc69* mutant harboring a construct *MC69p::MC69::HA* (*mc69*+*MC69::HA*) or a *MC69p::MC69::3xFLAG* (*mc69*+*MC69::3xFLAG*) to perform immunodetection of MC69::HA or MC69::3xFLAG proteins *in planta*, respectively. Both of *mc69*+*MC69::HA* and *mc69*+*MC69::3xFLAG* restored appressorial penetration and invasive growth in rice leaf sheath (Figure S3A and C), showing that the fusion proteins MC69::HA and MC69::3xFLAG are functional for pathogenicity. To clarify whether the MC69::HA and MC69::3xFLAG are expressed or not, *mc69*+*MC69::HA*- and *mc69*+*MC69::3xFLAG*-infected rice leaf sheath extracts were analyzed by SDS-PAGE gel blot analysis. We extracted total protein at 24 and 48 hours after leaf sheath inoculation. Note that at 24 hours after inoculation, most conidia develop appressoria but hyphal invasion is still limited, whereas at 48 hours extensive hyphal growth develops. Both of HA- and 3xFLAG-tagged MC69 were detected only faintly 24 hours after inoculation, but these proteins were abundant 48 hours after inoculation (Figure S3B and D) indicating that MC69 protein was indeed produced in invasive hyphae. Furthermore, we tried to express an *AVR* effector gene, *AVR-Pia* [18], [20] from the *MC69* promoter to see whether *AVR-Pia* avirulence function is supported by *MC69* promoter in rice plant harboring *Pia* R-gene. We hypothesized that only when *MC69* promoter allows expression of *AVR-Pia* in invasive hyphae, a sufficient amount of AVR-Pia protein would be translocated inside rice cells to be recognized by Pia, NBS-LRR-type cytoplasmic R-proteins [34]. We performed transformation of the *M. oryzae* isolate Ina86-137 (that lacks *AVR-Pia* and can infect rice cultivars possessing *Pia R*-gene) [20] with a construct *MC69p::AVR-Pia*. We used the wild-type stain (Ina86-137 WT) and a transformant harboring an intact copy of *AVR-Pia* (+*AVR-Piap::AVR-Pia*) [20] as negative and positive controls, respectively. In contrast with the Ina86-137 WT, +*AVR-Piap::AVR-Pia* and the transformants harboring *MC69p::AVR-Pia* (+*MC69p::AVR-Pia-1* and *-2*) failed to cause disease in the rice cultivar Sasanishiki possessing *Pia* (Figure S4A). Both Ina72-WT and the transformants successfully infected rice cultivar Shin No. 2 that lacks *Pia*, suggesting that their inability in infecting cv Sasanishiki was caused by *Pia*-*AVR-Pia* interactions and that the *MC69* promoter is expressed during invasive growth. Active transcription of the *AVR-Pia* in the transformants was confirmed by RT-PCR (Figure S4B) [20].

**Figure 3:**
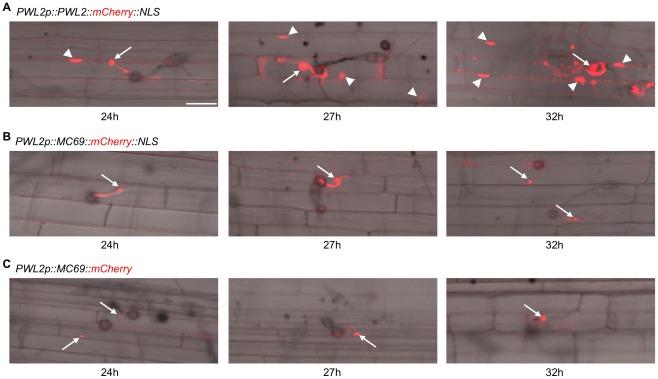
MC69::mCherry is not translocated into the rice cytoplasm. Merged DIC and mCherry images of rice leaf sheath cells infected by *M. oryzae* Sasa2 strain harboring (A) *PWL2p::PWL2::mCherry::NLS*, (B) *PWL2p::MC69::mCherry::NLS*, and (C) *PWL2p::MC69::mCherry* 24, 27 and 32 h after inoculation as observed by confocal laser scanning microscopy. Arrows indicate BICs and triangles indicate rice nuclei. Pinhole setting is 240 µm for all panels. Scale bar = 20 µm.

Khang *et al.* (2010) reported that secreted fluorescent effectors preferentially accumulate in biotrophic interfacial complexes (BICs) at the invasive hyphae-rice cell interface [11]. By fusing nuclear localization signal (NLS) to the fluorescent effectors to facilitate visualization of translocation, they also showed that the two BIC-localized secreted proteins, PWL2 and BAS1 were translocated into rice cytoplasm [11]. To test translocation of MC69::mCherry in rice cells, we added a modified small NLS from simian virus large T-antigen [35] at the C terminus of the MC69::mCherry fusion downstream of the *PWL2* promoter (*PWL2p::MC69::mCherry::NLS*) and transformed *M. oryzae* strain Sasa2 with the construct. A transformant *M. oryzae* harboring *PWL2p::PWL2::mCherry::NLS* was used as positive control. PWL2::mCherry::NLS exhibited significant fluorescence in BIC and in nuclei of invaded host cells at successful infection sites 24, 27 and 32 hours after inoculation (Figure 3A) whereas MC69::mCherry::NLS did not show fluorescence in nuclei of the invaded rice cells, but showed weaker fluorescence in BIC than that of PWL2::mCherry::NLS (Figure 3B). To eliminate the possibility that the NLS influences BIC localization, a transformant strain harboring *PWL2p::MC69::mCherry* was inoculated to rice leaf sheath. The result showed that MC69::mCherry was also detected in the BIC (Figure 3C). In addition, we observed mCherry fluorescence with different pinhole settings to compare the signals in the BIC among the three strains 27 hours after inoculation. The result showed that BIC accumulation signals of MC69::mCherry::NLS and MC69::mCherry were significantly weaker than that of PWL2::mCherry::NLS (Figure S5). These results suggest that the MC69 does not translocate into the infected rice cells, but localizes in BIC, however the accumulation level of MC69 in BIC is significantly lower than that of PWL2.

### Two cysteine residues are essential for MC69 function

**Figure 4:**
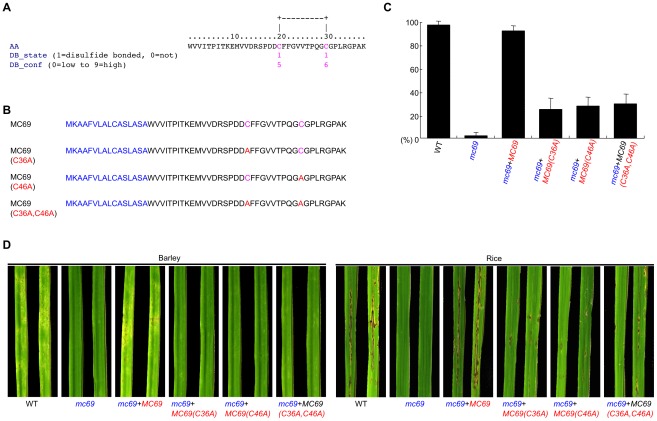
Two cysteine residues are important for MC69 virulence activity. (A) DISULFIND (http://disulfind.dsi.unifi.it/) output of the mature form of MC69 protein. (B) Amino acid sequences of disulfide mutants of MC69. The predicted signal peptide is indicated in blue. (C) Appressorial penetration by *M. oryzae* wild-type strain Ina72 (WT) and transformants with various versions of MC69. Mean percentage of invasion in rice (cv. Shin No. 2) leaf sheath cells 30 h after inoculation is presented. Four replicates of ∼50 appressoria were counted for each observation. (D) Blast symptoms caused by WT, the *mc69* mutant (*mc69*), the *MC69*-, *MC69(C36A)*-, *MC69(C46A)*- and the *MC69(C36A,C46A)*-re-introduced strains [*mc69*+*MC69*, *mc69*+*MC69(C36A)*, *mc69*+*MC69(C46A)* and *mc69*+*MC69(C36A,C46A)*] on barley (cv. Nigrate) and on rice (cv. Shin No. 2) 4 and 7 days after inoculation, respectively.

MC69 homologs were found in other filamentous fungi *Colletotrichum orbiculare* (AB669186), *Glomerella graminicola* (EFQ29542), *Verticillium albo-atrum* (EEY15898), *V. dahliae* (EGY20943), *Neurospora crassa* (XP_965292), *N. tetrasperma* (EGO52621), *Myceliophthora thermophila* (XP_003659994), *Podospora anserina* (XP_00190740), *Grosmannia clavigera* (EFX05010), *Fusarium oxysporum* (EGU75378), *Gibberella zeae* (XP_388669), *Trichoderma atroviride* (EHK44387), *T. virens* (EHK23962), *Metarhizium acridum* (EFY93067), *M. anisopliae* (EFY97094) and *Cordyceps militaris* (EGX95034) (Figure S6 and S7). However, these amino acid sequences did not contain known domains/motifs that would allow the prediction of their function. Nevertheless, MC69 homologs contain two conserved cysteine residues in the mature protein region C-terminal to the signal peptide (Figure S6). A software DISULFIND (http://disulfind.dsi.unifi.it/; [36]) predicted that the two cysteine residues in mature MC69 can form a disulfide bond (Figure 4A). To test whether these cysteines are necessary for MC69 function, mutant alleles of *MC69* were generated in which each or both of C36 and C46 were replaced with alanine (Figure 4B). Mutant alleles with one amino acid replacement (MC69(C36A); MC69(C46A)) or two replacements (MC69(C36A,C46A)) were expressed in the *mc69* mutant (*mc69*+*MC69(C36A)*, *mc69*+*MC69(C46A)* or *mc69*+*MC69(C36A,C46A)*). In all cases, appressorial penetration rate and blast symptoms on barley and rice were slightly restored, but still significantly reduced as compared to the wild type (Figure 4C and D). In addition, we further analyzed invasive growth rating of the 50 appressorial penetration sites. Infectious growth of *mc69*+*MC69(C36A)*, *mc69*+*MC69(C46A)* and *mc69*+*MC69(C36A,C46A)* within the inner epidermal tissue was slightly restored as compared to the *mc69* mutant (Figure S8). These results indicate that C36 and C46, presumably involved in disulfide bond, are necessary for MC69 to exert its pathogenicity in *M. oryzae*.

**Figure 5:**
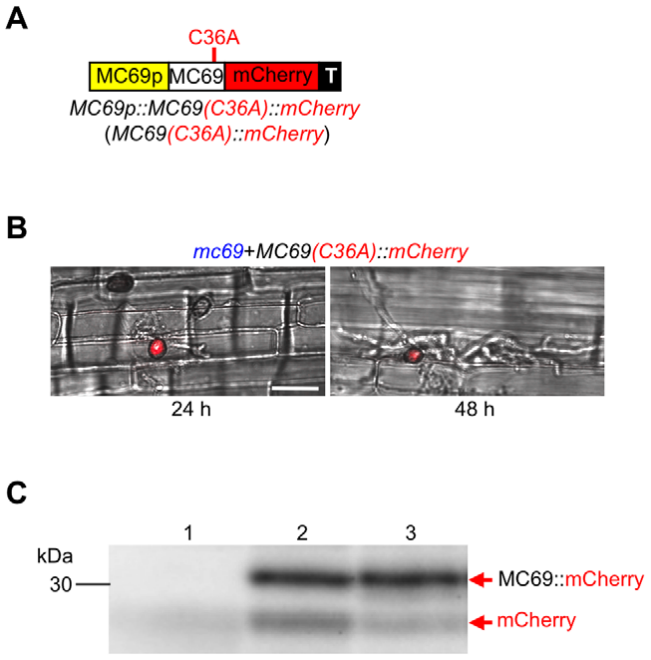
Secretion of MC69(C36A)::mCherry and MC69::mCherry fusion proteins. (A) Schematic diagram of MC69(36A)::mCherry fusion protein expression construct. (B) Merged DIC and mCherry images of rice leaf sheath cells infected by the MC69(C36A)::mCherry-expressing transformants 24 h and 48 h after inoculation. Scale bar = 20 µm. (C) Western blot probed with an anti-DsRed antibody. Samples were loaded as follows: lane 1, culture filtrate from mCherry-expressing strain; lane 2, culture filtrate from MC69::mCherry-expressing strain; lane 3, culture filtrate from MC69(C36A)::mCherry-expressing strain.

To see whether C36 and C46 are important for MC69 secretion/localization, spatial localization of the MC69(C36A) protein was tested by transforming *mc69* mutant with a construct *MC69p::MC69(C36A)::mCherry*, resulting in *mc69*+*MC69(C36A)::mCherry* (Figure 5A). We inoculated conidial suspension of the strain to rice leaf sheath to observe the mCherry fluorescence in the infected tissue. The mCherry fluorescence was detected in appressoria but not in the invaded hyphae 24 and 48 hours after inoculation (Figure 5B). The result suggests that the MC69(C36A)::mCherry protein was secreted into the plant and diffused below the detection limit like MC69::mCherry (Figure 2F and 5B). To clarify whether the MC69::mCherry and MC69(C36A)::mCherry are secreted or not, extracellular proteins secreted by *Magnaporthe* after liquid culture were analyzed by SDS-PAGE gel blot analysis. We used the wild-type strain expressing mCherry under *MC69* promoter (WT+*mCherry*; Figure 2B and C) as negative control. Western blot analysis (Figure 5C) revealed the presence of mCherry-tagged MC69 and mCherry-tagged MC69(C36A) in the culture medium. Faint signals of cleaved mCherry were observed as well for the transformants *mc69*+*MC69::mCherry* and *mc69*+*MC69(C36A)::mCherry*. The molecular weight (MW) of the fusion proteins was around 30 kDa, in line with the predicted MW of mature MC69::mCherry. These data strongly suggest that both of MC69 and MC69(C36A) are secreted to the medium, and C36 is not important for MC69 secretion. It could be possible that mutation of the Cys residues may impact pathogenicity by reducing the stability of the protein after secretion *in planta*.

### *MC69* is commonly required for pathogenicity in *M. oryzae*

**Figure 6:**
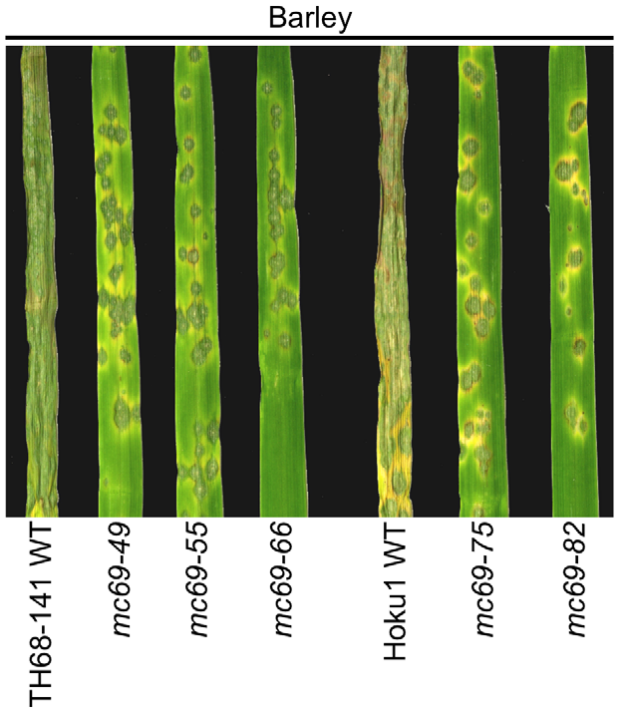
*MC69* is required for pathogenicity of other two different Japanese field isolates TH68-141 and Hoku1. Conidial suspension of wild-type strain TH68-141 (TH68-141 WT), the three independent *mc69* mutants (*mc69-49*, *mc69-55* and *mc69-66*), wild-type strain Hoku1 (Hoku1 WT) and the two independent *mc69* mutants (*mc69-75* and *mc69-82*) were inoculated on barley (cv. Nigrate) leaves and incubated for 5 days.

To investigate whether pathogenicity function of MC69 is conserved in *M. oryzae*, we produced *mc69* disruptants in other two Japanese field isolates of TH68-141 and Hoku1, in addition to the isolate Ina72. We used the *MC69* knockout vector used for Ina72 to generate *mc69* disruptants of both TH68-141 and Hoku1 isolates. Generated *mc69* disruptants of the two isolates showed a reduced pathogenicity on barley leaves as compared to the wild type strains (Figure 6), indicating the importance of MC69 in virulence of TH68-141 and Hoku1.

**Figure 7:**
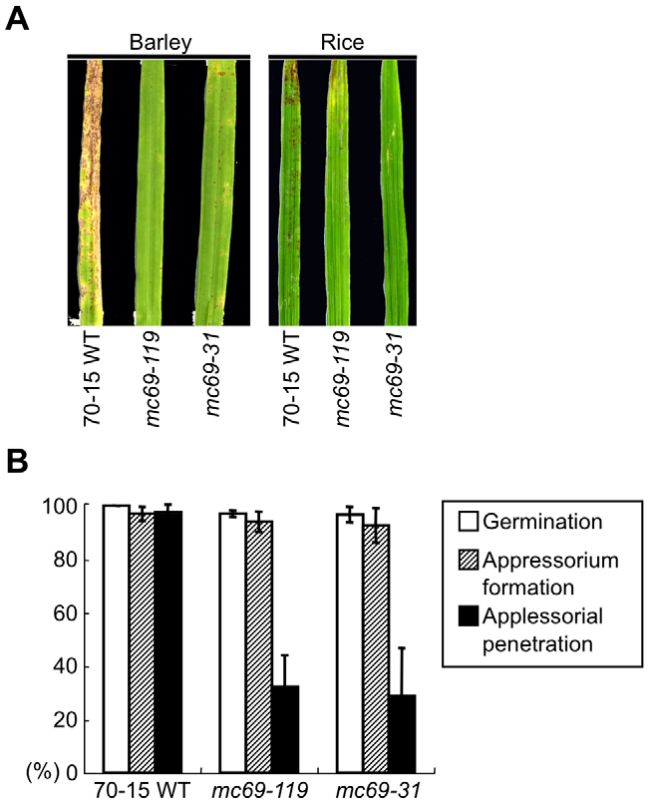
*MC69* is necessary for appressorial penetration and pathogenicity of a laboratory strain 70-15. (A) *MC69* is required for pathogenicity of *M. oryzae* strain 70-15. Conidial suspension of the wild-type strain 70-15 (70-15 WT) and the *mc69* mutants (*mc69-119*, *-31*) were inoculated on barley (cv. Nigrate) and rice (cv. Shin No. 2) leaves, and incubated for 7 days. (B) Germination, appressorium formation and appressorial penetration of 70-15 WT and the *mc69* mutants. The ratio of germination was calculated as the mean percentage of conidia germinated after 32 h on rice (cv. Shin No. 2) leaf sheath cells. The mean percentage of appressorium formation on rice leaf sheath cells among the germinated conidia is presented. Three replicates of ∼50 conidia were counted for each observation. The mean percentage of appressorial penetration by the *mc69* mutants is presented 32 h after inoculation. Standard errors are indicated by the vertical bars.

The whole genome sequence of 70-15, a well-studied laboratory strain of *M. oryzae*, was published [23]. We found that 70-15 showed poor virulence as compared to the Japanese strains in the previous study. It caused intermediate responses in all of the 13 tested rice cultivars: infection caused reddish lesions of various sizes, but they did not further develop into typical susceptible brown spindle-shaped necrotic lesions [20]. To investigate whether *MC69* is required for pathogenicity in 70-15, *MC69* gene disruption analysis was performed in the 70-15 background (Figure S1). Two independent *MC69*-KO lines (*mc69-119* and *mc69-31*) and wild-type 70-15 were sprayed onto barley cotyledons and rice leaves. The barley and rice infected by *mc69* mutants showed much weaker symptoms as compared to the 70-15-infected plants (Figure 7A), indicating the importance of MC69 in pathogenicity of 70-15. Appressorial penetration rates of the mutants in rice leaf sheath cells were significantly lower than that of 70-15 but the rates of germination and appressorium formation were same with the wild type (Figure 7B). In addition, we further analyzed invasive growth rating of 50 appressorial penetration sites. Infectious growth of the *mc69* mutants within the inner epidermal tissue was restricted as compared to the wild type (Figure S9). However, the colony growth and conidiation of the mutants on oatmeal agar media were similar to the wild type (Figure S2C and D). Thus, the *mc69* mutants of 70-15 have a defect in appressorial penetration and development of blast symptoms, which is similar to the phenotype of the *mc69* disruptants of Ina72. These results suggest that MC69 is commonly required for appressorial penetration and subsequent colonization in various *M. oryzae* strains.

### An ortholog of *MC69* is required for pathogenicity of *Colletotrichum orbiculare*

**Figure 8:**
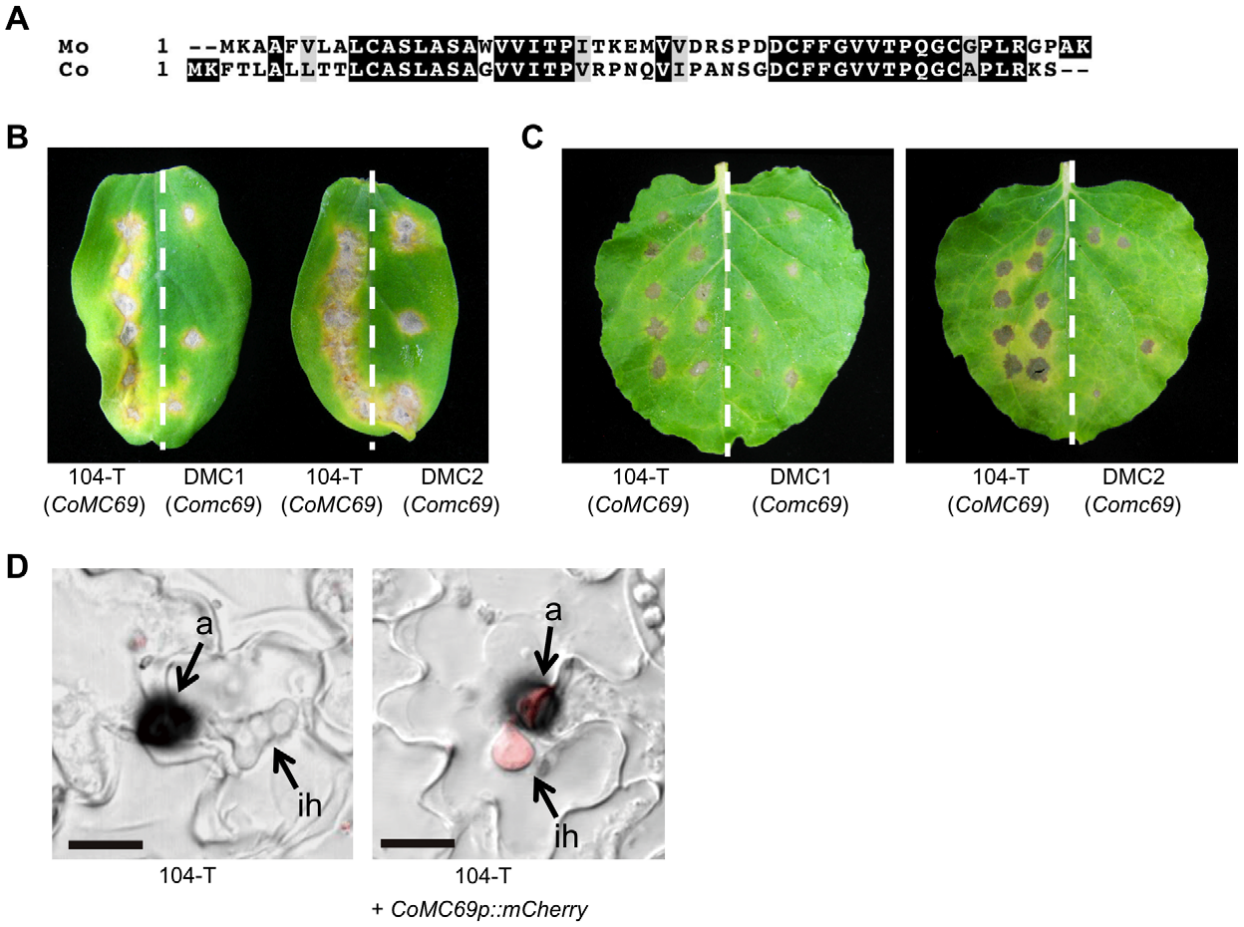
*CoMC69* is involved in fungal pathogenicity of *C. orbiculare*. (A) Sequence alignment of MC69 between *M. oryzae* (Mo) and *C. orbiculare* (Co). Amino acid sequences were aligned using Clustal W program [52]. Identical amino acids are indicated as white letters on a black background. Similar residues are shown by gray background. Gaps introduced for alignment are indicated by hyphens. (B) Pathogenicity test of the *Comc69* mutants on cucumber. Conidial suspensions were inoculated on detached cotyledons of cucumber (*Cucumis sativa*). On the left half of the cotyledons, the wild-type strain 104-T was inoculated as positive control. On the right half, the *Comc69* strains (DMC1 and DMC2) were inoculated. Inoculated cotyledons were incubated for 7 days. (C) Pathogenicity test of the *Comc69* mutants on *N. benthamiana*. On the left half of the detached leaves of *N. benthamiana*, the strain 104-T was inoculated as positive control. On the right half, the *Comc69* strains (DMC1 and DMC2) were inoculated. Inoculated leaves were incubated for 7 days. (D) mCherry-based reporter assay for expression of the *CoMC69* gene. Conidia from the *C. orbiculare* strain carrying the *CoMC69* promoter-*mCherry* fusion gene (*CoMC69p::mCherry*) was inoculated onto the lower surfaces of cucumber cotyledons, and the inoculated plant was incubated for 4 days. a, appressorium; ih, intracellular hypha. Scale bars = 10 µm.

The importance of MC69 in *M. oryzae* raised a possibility that *MC69* orthologs are also involved in pathogenicity of other fungal pathogens. To assess this point, we investigated whether *MC69* ortholog is involved in pathogenicity of the cucumber anthracnose fungus *C. orbiculare* (Figure S6). A gene homologous to *MC69* was isolated from *C. orbiculare* in this study. The isolated gene, designated *CoMC69*, comprises 220 bp interrupted by an intron and encodes a predicted protein of 54 amino acids (Figure 8A). Intron/exon organization in *MC69* orthologs in filamentous fungi indicated that most of them have one intron only followed by an exon (140–156 bp) except for the genes in *T. virens* and *Gibberella zeae* (Figure S10). First exons in all genes encode a common region containing two conserved cysteine residues in the mature proteins (Figure S6).

To investigate whether *CoMC69* is involved in the pathogenicity of *C. orbiculare*, we produced *CoMC69* disruption mutants. The plasmid pCBGDMC69 was designed to replace the *CoMC69* gene in the wild-type strain 104-T through double crossover homologous recombination (Figure S11A and B).

The colony morphology and conidiogenesis of *Comc69* mutants grown on PDA medium were similar to that of 104-T (Figure S11C and data not shown). We next investigated their pathogenicity on host cucumber leaves. Conidial suspensions from the *Comc69* mutants were spotted on detached cucumber leaves and incubated for 7 days. The *Comc69* mutants exhibited clear reduction in lesion development in comparison with the wild-type strain 104-T (Figure 8B). *C. orbiculare* 104-T is able to infect *Nicotiana benthamiana*, which is not closely related to cucumber [37]. The *Comc69* mutants also exhibited reduced pathogenicity on *N. benthamiana* (Figure 8C). These results indicate that *CoMC69* is required for pathogenicity of *C. orbiculare*, suggesting conserved roles of the MC69 proteins in pathogenicity of both *M. oryzae* and *C. orbiculare*. To investigate the gene expression of *CoMC69* in plant infection of *C. orbiculare*, we generated *C. orbiculare* strains carrying a reporter plasmid containing the 1.4 kb 5′ upstream region of *CoMC69* fused with *mCherry*. As a result, we found the mCherry fluorescence in appressoria and primary intracellular hyphae of the transgenic *C. orbiculare*, indicating the expression of *CoMC69* in the plant infection stage of *C. orbiculare* (Figure 8D).

## Discussion

In this study, we show that MC69, a novel secreted protein of *Magnaporthe oryzae*, is essential for successful appressorial penetration and blast symptom development in rice and barley cultivars. The *MC69* gene (MGG_02848.6) resides on chromosome VII of the *M. oryzae* genome. The MC69 protein comprises 54 amino acids and is predicted to harbor a putative N-terminal secretion signal peptide (Figure 4B). *MC69* seems to be a solitary gene without any paralogs in the genome. It lacks known sequence motifs associated with enzymatic function. Although *MC69* homologs were found in other filamentous fungi (Figure S6), their functions are also not known. Expression of *MC69* was observed in mycelia, conidia and all stages of infection (Figure 2). The *mc69* disruptants were unable to invade plant cells to establish compatible interaction with the host plant (Figure 1, 6 and 7). However in *mc69* mutants, other phases of infection-related development such as conidial germination and appressorium formation were unaffected (Figure 1B and 7B).

MC69 of *M. oryzae*, which is predicted to comprise 38 amino acids after cleavage of the signal peptide, contained no known functional domains. Therefore it is unlikely that MC69 has an enzymatic function. The two cysteine residues (C36 and C46) conserved among the MC69 homologs may be involved in disulfide bridge formation and were shown to be important for pathogenicity function of MC69 (Figure 4 and S6). Pep1 is a novel effector protein from the corn smut fungus *Ustilago maydis* that is essential during penetration. Disruption mutants of *pep1* are not affected in saprophytic growth and develop normal infection structures, but are arrested during the penetration of epidermal cells of maize leaves. In addition, two of the four cysteine residues in Pep1 were shown to be essential for the virulence function [38]. The authors consider that the importance of two of the four cysteine residues for secretion of Pep1 to make a compact structure with disulfide bridge structure of Pep1 [38]. To address this possibility we observed localization of MC69::mCherry and MC69(C36A)::mCherry in *M. oryzae*. We were unable to detect red fluorescence in infectious hyphae and the fusion proteins were detected in the culture filtrate of both strains (Figure 2 and 5). These results indicated that the substitution of cysteine residues of MC69 did not affect secretion, but affected pathogenicity of *M. oryzae*.

On the other hand, disruption of a total of 77 secreted protein genes in *M. oryzae* did not affect its pathogenicity within our experimental condition. Since there is no systematic bias in our selection of secreted protein genes for disruption, we extrapolate that 77/78 = 99% of secreted protein genes do not show clear reduction in pathogenicity even after knockout. Several secreted avirulence (*AVR*) effector genes have been isolated from *M. oryzae*, including PWL effectors [16], [19], *AVR-Pita* [13], [14], *AVR1-CO39* [15], *AVR-Piz-t* [17], *AVR-Pia*, *AVR-Pii* and *AVR-Pik/km/kp* [18], [20], but the virulence functions of the genes are still unknown. In fact, the AVR-Pita effector is dispensable for virulence on rice [14], [39]. According to our results and available information on *M. oryzae* AVR effectors, we hypothesize two mutually non-exclusive possibilities: (1) virulence contribution of most of effectors is too small to be detected by conventional assays; (2) effectors have redundant activities and more than one effector participate in the same virulence pathway.

A recent report of *M. oryzae* indicates that the fungus overcomes the first line of defense (PAMP-Triggered Immunity) by secreting an effector protein, Slp1 during invasion of new rice cells [21]. There are several reports for secreted effectors of other fungal pathogens. Pep1 of *U. maydis* was described above. Several hydrophobins or repellent genes that encode secreted proteins of *U. maydis* were examined for their roles in virulence. Single knock-outs of these genes did not affect virulence, but a double knockout of the repellent-encoding gene *Rsp1* and *Hum3* (a gene encoding a protein containing both, a hydrophobin domain and a repellent region) were arrested at an early stage of penetration. This indicates that Rsp1 and Hum3 are effectors with a partly redundant virulence function during the early stages of infection [40]. We speculate a similar situation may occur in *M. oryzae*. It would be a good way to focus on effector candidates exhibiting higher similarities and knockout or silence multiple genes simultaneously, to identify the multiple effectors that act redundantly.

We showed that *MC69* was required for pathogenicity of the additional three strains of *M. oryzae* in addition to the strain Ina72, indicating conserved roles of MC69 in *M. oryzae*. To investigate whether the pathogenicity function of the *MC69* ortholog in the well-studied dicot fungal pathogen, we isolated an ortholog, *CoMC69* from the cucumber anthracnose fungus, *Colletotrichum orbiculare*. Notably, in *C. orbiculare* the deletion of *CoMC69* reduced pathogenicity on the hosts cucumber and *Nicotiana benthamiana* leaves (Figure 8).

Phylogenetic analyses were performed with *M. oryzae* MC69 (MoMC69), with 16 homologs from other phytopahogenic (*C. orbiculare*, *Glomerella graminicola*, *Verticillium albo-atrum*, *V. dahliae*, *Grossmannia clavigera*, *Fusarium oxysporum* and *Gibberella zeae*), entomopathogenic (*Metarhizium acridum* and *M. anisopliae*), caterpillar killer (*Cordyceps militaris*), fungal parasite (*Trichoderma atroviride* and *T. virens*) or saprophytic (*Neurospora crassa*, *N. tetrasperma*, *Myceliophthora thermophila* and *Podospora anserina*) fungi (Figure S7). MoMC69 and CoMC69 are closely related to the Verticillium wilt pathogens *V. albo-atrum*, *V. dahliae* and the cereal plants anthracnose fungus *Glomerella graminicola*. A conserved motif containing the two cysteine residues showed high homology among all MC69 homologs (Figure S6). Thus, it will be interesting to determine whether genes orthologous to *MoMC69* also contribute to the pathogenicity of various plant, fungus, entomo or caterpillar pathogenic fungi. However, *MC69* orthologs also occur in saprophytes suggesting that a possibility that the primary function of the protein is in relation to the structure or function of the fungus itself, and that the function must be intact for the fungus to succeed as a pathogen.

We generated transgenic rice overexpressing *MC69* to examine susceptibility to *M. oryzae* wild-type strain or the *mc69* mutant infection. However, overexpression of *MC69* in rice neither enhanced the pathogenicity of *M. oryzae* wild-type strain nor complemented the pathogenicity deficiency of the *mc69* mutant (data not shown). We hypothesize three possibilities why overexpression of *MC69* did not affect *M. oryzae* wild-type strain and the *mc69* mutant infection. One possibility is that the localization of MC69 in the infection sites of *M. oryzae* in rice cells is important. We produced *M. oryzae* transformant harboring *PWL2p::MC69::mCherry*. After inoculation of the strain to the rice leaf sheath, mCherry fluorescence was detected in biotrophic interfacial complex (BIC) (Figure 3C). However, MC69::mCherry fusion protein expressed by *MC69* promoter was not detected in BIC (Figure 2F). It might be because *MC69* promoter activity was weaker than *PWL2* promoter activity or *PWL2* promoter leads MC69::mCherry to BIC accumulation but *MC69* promoter did not. To test these possibilities, *mc69* mutant expressing MC69::EGFP fusion protein downstream of *MC69* promoter has been produced because EGFP fluorescence was relatively stronger than mCherry fluorescence. When the transformant was inoculated to the rice leaf sheath, MC69::EGFP was shown to be accumulated to the BIC (data not shown). These results indicate that BIC localization of MC69 is important for virulence of *M. oryzae*. Therefore, ectopic overexpression of *MC69* in rice neither enhanced pathogenicity of wild-type strain nor complemented deficiency of *mc69* mutant of *M. oryzae* in trans because of the MC69 protein would not be localized in BIC. The second possibility is that post translational modification of MC69 protein in *M. oryzae* might be different from that *in planta* even though the secreted MC69::mChrerry protein shows an expected molecular size (Figure 5C). The third possibility is that MC69 affects the physiology of the fungus but does not directly affect the physiology of the plant so that expression of MC69 in rice did not complement the defect in *mc69* mutant of *M. oryzae*.

Khang *et al.* (2010) demonstrated that BIC-localized secreted proteins PWL2 and BAS1 were translocated into the rice cytoplasm but a secreted protein BAS4, which uniformly outlines the invasive hyphae, was not [11]. Interestingly, when *M. oryzae* transformants secreted fluorescent MC69 fusion protein during epidermal cell invasion, the fluorescent protein was observed in BICs (Figure 3). To investigate whether this feature is specific to MC69 or not, we generated *M. oryzae* strains expressing fluorescence-labeled version of four putative secreted proteins (HMM14, MC55, GAS1 or GAS2) with NLS. HMM14 and MC55 are the secreted protein genes studied here (Table 1) and GAS1 and GAS2 are secreted protein genes involved in virulence of *M. oryzae* [28]. The generated strains were inoculated to rice leaf sheaths, and localization of each protein was investigated. The result showed that all four proteins were localized to the BICs, and both GAS1 and GAS2 were then translocated into the rice cytoplasm, which is similar to PWL2. By contrast, HMM14 and MC55 were not translocated into the rice cytoplasm like MC69 (data not shown). However, fluorescent signals of BIC accumulations of MC69::mCherry::NLS, MC69::mCherry, HMM14::mCherry::NLS and MC55::mCherry::NLS were significantly weaker than that of PWL2::mCherry::NLS, GAS1::mCherry::NLS and GAS2::mCherry::NLS (Figure 3, S5 and data not shown). These finding indicate that BIC accumulation level of secreted proteins might be important for translocation to the infected rice cells.

A virulence effector Slp1 sequesters chitin oligosaccharides to prevent PAMP-triggered immunity in rice, thereby facilitating rapid spread of the fungus within host tissue [21]. Slp1 contains two putative LysM domains, which have previously been shown to bind carbohydrates [41]. An effector known as Ecp6 that also contains LysM domains was identified from a fungal pathogen *Cladosporium fulvum* that causes leaf mold of tomato [42]. Another effector AVR4 of *C. fuluvum* binds to chitin present in fungal cell walls and that, through this binding AVR4 can protect these cell walls against hydrolysis by plant chitinases [43]. Growth of the *Δpep1* mutants of *U. maydis* are arrested during penetration of the epidermal cell and elicit a strong plant defense response such as formation of large papillae, induction of strong cell wall autofluorescence, H_2_O_2_ accumulation and defense related gene expression [38]. We tried to elucidate the roles of MC69 by addressing differences in H_2_O_2_ accumulation and expression of defense related genes in rice infected by *mc69* mutant and wild type *M. oryzae*. However, results showed no difference between *mc69* and wild type so that we have no evidence that MC69 suppresses plant defense responses at the moment (data not shown). Taken together, we demonstrated that MC69 has a pathogenicity function (required for the fungus to be a pathogen), but its function has yet to be elucidated.

## Materials and Methods

### Fungal strains, medium and transformation

All isolates of *M. oryzae* used in this study are stored at the Iwate Biotechnology Research Center. Fungal strains used were the wild-type strains 70-15, Ina72, TH68-141, Hoku1, Sasa2 and Ina86-137 [20]. To obtain protoplasts, hyphae of *M. oryzae* strains were incubated for 3 days in 200 mL of YG medium (0.5% yeast extract and 2% of glucose, w/v). Protoplast preparation and transformation were performed as described previously [44]. Hygromycin- or bialaphos-resistant transformants were selected on plates with 300 µg ml^−1^ of hygromycin B (Wako Pure Chemicals, Osaka, Japan) or 250 µg ml^−1^ of bialaphos (Wako Pure Chemicals). *C. orbiculare* (Berk. & Mont.) Arx (syn. *C. lagenarium* [Pass.] Ellis & Halst.) strain 104-T (MAFF240422) was used as the wild-type strain. All *C. orbiculare* strains were maintained on 3.9% (w/v) PDA (Difco Laboratories, Detroit, MI) at 24°C. Preparation of protoplasts and transformation of *C. orbiculare* were performed according to a method described previously [45].

### SuperSAGE of cAMP-treated *M. oryzae* strain 70-15

Mycelia of *M. oryzae* were grown on oatmeal agar medium (30 g l^−1^ oatmeal, 5 g l^−1^ sucrose and 16 g l^−1^ agar). To enhance conidia formation, the fungus was first grown on oatmeal agar medium for 9 days at 25°C, and then exposed to Black Light Blue light (Toshiba FS20S/BLB 20W; Toshiba, Tokyo, Japan) for 4 days at 22°C, after aerial hyphae of the colonies had been washed away with sterilized distilled water. Conidia of *M. oryzae* were suspended in 50 mM cAMP to a final density of 1×10^6^ conidia ml^−1^. This suspension was then poured onto dialysis membranes (2.5 ml of suspension/25 cm^2^ membrane surface; Spectra/Por, cutoff 1,000 Da; Spectrum Medical Industries, Terminal Annez, LA) and incubated at 25°C in dark [25]. Total RNA was extracted from germinating conidia incubated for 6 h on dialysis membranes, as described below. 32 sheets of membranes containing the germinating conidia were crushed and homogenized in liquid nitrogen with mortar and pestle. The homogenate was transferred to a centrifuge tube containing 40 ml of TRI Reagent (SIGMA-ALDRICH, St. Louis, MO), homogenized by vigorous shaking and incubated at room temperature for 5 min. Then 8 ml of chloroform was added, homogenized by vigorous shaking for 15 sec and incubated at room temperature for 3 min. After centrifugation at 1000× g for 15 min at 4°C, the upper aqueous phase was transferred to a new centrifuge tube, and the total RNA was precipitated by the addition of 20 ml of isopropanol after incubation at room temperature for 10 min. The pellet was rinsed with 70% ethanol. SuperSAGE library was made from total RNA as described [46], [47]. Di-tag fragments were sequenced by the 454 FLX sequencer (454 Life Sciences). Each 26-bp tag sequence was used for BLASTN search against *M. oryzae* 70-15 genome sequence. A total of 23,491 tags to comprising 26-bp sequence were recovered. Number of tags for each of putative secreted protein genes of *M. oryzae* is given in Table S1.

### Plasmid construction

To construct the gene-disruption vector pGPSMC69-44, a 8.7-kb fragment containing the *MC69* gene amplified with the primers MC69S1 (5′- TTATGACGGGAGCACAGGCACAGCACAC-3′) and MC69AS1 (5′- TGGCCGACGTTGTGCTCTTTCAGTTCCT-3′) was cloned into pCR-XL-TOPO to generate pXLMC69 using the TOPO XL PCR Cloning Kit (Invitrogen, Carlsbad, CA). *MC69* was mutated using an adaptation of the TAG-KO method using pGPS-HYG-CAM [30], [31]. The pXLMC69 containing *MC69* was used as the target. An insertion was formed within the coding region of *MC69* (at 11 amino acids) in pXLMC69, which resulted in pGPSMC69-44 (Figure S1).

For complementation assay of an *mc69* mutant with *MC69*, a 5.7-kb fragment containing *MC69* was amplified with the primers NMU1 (5′-ATAAGAATGCGGCCGCTGATTCTCAATGCCCTCTGTCCTTT-3′; the NotI site is underlined) and MC69AS1. The PCR product was digested with NotI and XbaI (exists in the middle of the PCR product after 1.1-kb far from the poly A signal recognition site of *MC69*) to generate 3.1-kb fragment containing *MC69*, and ligated to the same restriction sites of which carries the bialaphos-resistant (bar) gene [48], creating pCB1531-MC69.

To substitute a cysteine residue at 36 amino acids in MC69 by alanine, single point mutation was introduced in plasmid pCB1531-MC69 using a primer BMC36AU4 (5′- CA*GGTCACC*AGACGACGCCTTCTTTGG -3′; mutation site is underlined and the BstEII site is indicated in italics). A 1.7-kb fragment containing a half 3′-terminal part of the *MC69* ORF and terminator was amplified with the primers BMC36AU4 and M13F (5′- CGCCAGGGTTTTCCCAGTCACGA-3′). The PCR product was digested with BstEII and XbaI, and exchanged to the BstEII/XbaI fragment of pCB1531-MC69, generating pCB1531-MC69(C36A) (Figure 4B). To substitute a cysteine residue at 46 amino acids in MC69 by alanine, single point mutation was introduced in plasmid pCB1531-MC69 using a primer MC46AU5 (5′-CGTCACGCCGCAAGGCGCCGGGTATGTTCTGGG-3′; mutation site is underlined). A 1.7-kb fragment was amplified with the primers MC46AU5 and M13F, and the PCR product was used as a template for another PCR with the primers BMC46AU4 (5′- CA*GGTCACC*AGACGACTGCTTCTTTGGTGTCGTCACGCCGCAAGGCGCCGG-3′; mutation site is underlined and the BstEII site is indicated in italics) and M13F. The PCR product was digested with BstEII and XbaI, and exchanged to the BstEII/XbaI fragment of pCB1531-MC69, generating pCB1531-MC69(C46A) (Figure 4B). To substitute two cysteine residues at 36 and 46 amino acids in MC69 by alanine, double point mutations were introduced in plasmid pCB1531-MC69 using a primer BMC36&46AU4 (5′- CA*GGTCACC*AGACGACGCCTTCTTTGGTGTCGTCACGCCGCAAGGCGCCGG-3′; mutation sites are underlined and the BstEII site is indicated in italics). A 1.7-kb fragment was amplified with the primers MC46AU5 and M13F, and the PCR product was used as a template for another PCR with the primers BMC36&46AU4 andM13F. The PCR product was digested with BstEII and XbaI, and exchanged to the BstEII/XbaI fragment of pCB1531-MC69, generating pCB1531-MC69(C36A,C46A) (Figure 4B).

For construction of the *MC69*-*EGFP* gene fusion vector pCB1531-MC69-EGFP, a 1.7-kb fragment containing *MC69* gene was amplified with the primers NMU1 and XMG5L1 (5′-G*CTCTAGA* CCACCACCACCACCTTTGGCAGGTCCGCGAAGAGGG-3′; XbaI site is indicated in italics) which was designed with five glycine codons (underlined) as a spacer peptide between MC69 and EGFP. The PCR product encoding MC69-Gly_5_ was digested with NotI and XbaI, and exchanged to the NotI/XbaI fragment of the *Tef* promoter in pBAGFP [49], generating pCB1531-MC69-EGFP. For construction of the *MC69*-*mCherry* gene fusion vector pCB1531-MC69-mCherry, a 0.7-kb mCherry cDNA fragment was amplified with the primers XmU1 (5′- GCTCTAGACATGGTGAGCAAGGGCGAGG-3′; XbaI site is underlined) and BmL1 (5′- CGGGATCCTACTTGTACAGCTCGTCCAT-3′; BamHI site is underlined) using pmCherry (Clontech, Mountain View, CA) as a template. The PCR product was digested with XbaI and BamHI, and exchanged to the XbaI/BamHI fragment of EGFP cDNA in pCB1531-MC69-EGFP, generating pCB1531-MC69-mCherry (Figure 2D), a 1.6-kb fragment of *MC69* promoter (*MC69p*) and *MC69(C36A)* ORF was amplified with the primers NMU1 and XMG5L1. The PCR product was digested with NotI and XbaI, and exchanged to the NotI/XbaI fragment of *MC69p*-*MC69* in pCB1531-MC69-mCherry, generating pCB1531-MC69(C36A)-mCherry (Figure 5A). A 1.4-kb fragment of *MC69p* was amplified with the primers NMU1 and XMpL2 (5′- GCTCAGACCTTCGTAGGCCTGGAACGAGACGCTTCC-3′; XbaI site is underlined). The PCR product was digested with NotI and XbaI, and exchanged to the NotI/XbaI fragment of *MC69p*-*MC69* in pCB1531-MC69-mCherry, generating pCB1531-MC69p-mCherry (Figure 2A).

A 0.6-kb fragment of *PWL2* promoter was amplified the primers Ppwl2-5′ (5′-GAGGAGAAGCGGCCGCGTTAACAACGCGGTGTAAAGATTC-3′; NotI site is underlined) and Ppwl2-3′ (5′-GAGAGGAGAAGGATCCACTAGTTCTAGATTTGAAAGTTTTTAATTTTAAAAAGAGATTTTCCGAG-3′; BamHI-SpeI-XbaI sites are underlined). The PCR product was digested with NotI and BamHI, and exchanged to the NotI/BamHI fragment of Tefp-EGFP in pBAGFP [49], generating pCB-Ppwl2. mCherry cDNA fragment was amplified with primers mCherry-N (5′-GAGAGGAGAAGGATCCAGATCTCTCGAGACCATGGTGAGCAAGGGCGAGGAG-3′; BamHI-BglII-XhoI sites are underlined) and mCherry-C (5′-GAGAGGAGAAGAATTCGCTAGCGTCGACCTTGTACAGCTCGTCCATG-3′; EcoRI-NheI-SalI sites are underlined). The PCR product was digested with BamHI and EcoRI, and introduced into pCB-Ppwl2, to produce pCB-Ppwl2-mCherry. pCB-Ppwl2-mCherry was digested with SalI, fill in with Klenow fragment and performed self-ligation, generating pCB-Ppwl2-mCherry-stop. A modified SV40 NLS-coding double stranded fragment [35] was produced annealing with the oligos mSV40NLS (5′-TCGACGGTCCAGGTGGAGCTGGACCAGGTAGAAAGAGGCCACCAAAGAAAAAGAGAAAGGTAGATTATGGAGCTTAAG-3′; SalI protruding end is underlined) and c-mSV40NLS (5′-AATTCTTAAGCTCCATAATCTACCTTTCTCTTTTTCTTTGGTGGCCTCTTTCTACCTGGTCCAGCTCCACCTGGACCG-3′; EcoRI protruding end is underlined). The annealed product was introduced into pCB-Ppwl2-mCherry, to produce pCB-Ppwl2-mCherry-NLS. From 1 µg of total RNA of cAMP-treated *M. oryzae* strain 70-15, single-stranded cDNA was synthesized by using oligo(dT) primer and ReverTra Ace reverse transcriptase (Toyobo, Osaka, Japan). A 0.4-kb *PWL2* cDNA fragment was amplified from the total cDNA as a template with the primers PWL2-N (5′-GAGAGGAGAATCTAGAAAAATGAAATGCAACAACATCATCCTC-3′; XbaI site is underlined) and PWL2-C (5′-GAGAGGAGAAGGATCCCATAATATTGCAGCCCTCTTCTC-3′; BamHI site is underlined). The PCR product was digested with XbaI and BamHI, and introduced into pCB-Ppwl2-mCherry-NLS, generating pCB-Ppwl2-PWL2-mCherry-NLS (Figure 3A). A 0.2-kb *MC69* cDNA fragment was amplified from the total cDNA with the primers XMU2 (5′-GCTCTAGAAAATAAAAATGAAGGCCGCT-3′; XbaI site is underlined) and XML1 (5′-CCGCTCGAGTTTGGCAGGTCCGCGAAGAGGGCCGC-3′, XhoI site is underlined). The PCR prodct was digested with XbaI and XhoI, and introduced into pCB-Ppwl2-mCherry-NLS and pCB-Ppwl2-mCherry-stop, to produce pCB-Ppwl2-MC69-mCherry-NLS and pCB-Ppwl2-MC69-mCherry, respectively (Figure 3B and C).

To make the *MC69*-*HA* gene fusion vector pCB1531-MC69-HA, HA-tagged full cDNA of *MC69* (*MC69HA*) was amplified from the total cDNA with the primers XMU2 and BMHAL1 (5′- CGggatccTCA*AGCATAATCTGGAACATCGTATGGATA* ACCACCTTTGGCAGGTCCGCGAAGAGGGCCGC-3′; BamHI site and HA tag sequence are indicated in lower cases and italics, respectively) which was designed with two glycine codons (underlined) as a spacer peptide between MC69 and HA tag. The PCR product was digested with XbaI and BamHI, and exchanged mCherry gene at the same sites of pCB1531-MC69p-mCherry, generating pCB1531-MC69-HA (Figure S3). *MC69-3xFLAG* gene fusion construct pUC57-MC69-3xFLAG was custum-synthesized (GenScript, Piscataway, NJ). *MC69-3xFLAG* was amplified from pUC57-MC69-3xFLAG with the primers SMU2 (5′-GACTAGTGAAAATAAAAATGAAGGCCGCTTTCGTTCTCGC-3′; SpeI site is underlined) and BFL1 (5′- CGGGATCCTCACCCATCATGATCCTTGTAATCG-3′; BamHI site is underlined). The PCR product was digested with SpeI and BamHI, and exchanged mCherry gene at XbaI and BamH sites of pCB1531-MC69p-mCherry, generating pCB1531-MC69-3xFLAG (Figure S3).

For construction of the *MC69p::AVR-Pia* epression vector pCB1531-MC69p-AVR-Pia, a 0.3-kb fragment containing *AVR-Pia* gene was amplified from pCB1004-pex22 [20] with the primers XP22U2 (5′-GCTCTAGACAAAATGCATTTTTCGACAATTTTC-3′; XbaI site is underlined) and BP22L2 (5′-CGGGATCCTAGTAAGGCTCGGCAGCAAGCC-3′; BamHI site is underlined). The PCR product was digested with XbaI and BamHI, and exchanged mCherry gene at the same sites of pCB1531-MC69p-mCherry, generating pCB1531-MC69p-AVR-Pia (Figure S4).

*CoMC69* was isolated from genome of *C. orbiculare* 104-T by PCR using degenerate primers designed in amino acid sequences of *MC69* homologs in fungal pathogens including *C. graminicola*. To construct the gene replacement vector pGDCOMC69, the 3.0-kb fragment containing the 5′ flanking region of *CoMC69* was amplified by PCR with the primers COMC5S (5′-ATAAGAATGCGGCCGCCCAGTGCTTTGTCATGTTGC-3′; NotI site is underlined) and COMC5AS (5′-CCCAAGCTTCGCTGGTTGCGAAGAATGCG-3′; HindIII site is underlined). The amplified fragment was digested with NotI and HindIII, and introduced into pCB1636 [48], which contained the *hph* gene, to produce plasmid pCB5MC69. The 3-kb fragment that contained the 3′ flanking region of *CoMC69* was amplified by PCR with the primers COMC3S (5′-GAAGGGCCCCCGGTCACCACGCATGTGTGATACG-3′; ApaI site is underlined) and COMC3AS (5′-GGGGTACCACGTGTGCACTCTTAAGGAG-3′; KpnI site is underlined). The amplified fragment was digested with ApaI and KpnI, and introduced into pCB5MC69 to produce pGDCOMC69 (Figure S11A). To generate the reporter construct pBATCoMC69pro-mCherry, the 1.4 kb 5′ upstream region of *CoMC69* and *mCherry* were amplified using PCR with the two primer sets, (i) CoMC69pro-NotI-f (5′-ATAAGAATGCGGCCGCGTCTTTCGTCTTTTCGGTCT-3′; NotI site is underlined) and CoMC69pro-BamHI-r(c) (5′-CGGGATCCCGTGTCGATGTATTTGTTGTG-3′; BamHI site is underlined), and (ii) mCherry-BamHI-f (5′-GCGGATCCATGGTGAGCAAGGGCGAGGAGGATAAC-3′; BamHI site is underlined) and mCherry-EcoRI-r(c) (5′-CCGGAATTCTTACTTGTACAGCTCGTCCATGCC-3′; EcoRI site is underlined), respectively. The amplified fragments were introduced into each corresponding site of pBAT [49], resulting in pBAT-CoMC69pro-mCherry.

### Pathogenicity assays

Barley leaf and rice leaf inoculation were performed as follows: conidial suspension (1×10^5^ conidia ml^−1^) containing Tween 20 (0.01% in final concentration) was sprayed onto susceptible barley cotyledons (cv. Nigrate) and rice seedlings (cv. Shin No. 2 or cv. Sasanishiki) of the fourth leaf stage. Inoculated plants were placed in a dew chamber at 27°C for 24 h in the dark, and then transferred to the growth chamber with a photoperiod of 16 h. A rice leaf sheath inoculation test was performed according to the method described previously [50]. To investigate the function of appressorium-mediated penetration of the inner epidermal tissue of rice leaf sheath, penetration hyphae were stained with lactophenol-trypan blue and destained in saturated chloral hydrate as described previously [51]. Invasive growth rating of the 50 appressorial penetration sites in rice leaf sheath cells were scored 32 h after inoculation. Invasive growth were classified into 4 levels: Level 1, invasive hypha length is shorter than 10 µm with no branch; Level 2, invasive hyphae length is 10–20 µm with 0–2 branches; Level 3, invasive hyphae length is longer than 20 µm and/or with more than 2 branches within one cell; Level 4, invasive hyphae are spread more than one cell (Figure 1D). To test fungal pathogenicity of *C. orbiculare*, conidial suspensions of tested *C. orbiculare* strains (approximately 5×10^5^ conidia/ml) were spotted onto detached leaves of cucumber or *N. benthamiana*.

### Confocal laser-scanning microscopy

Germinated conidia and appressoria were observed on glass coverslips, and invaded hyphae were observed in epidermal cells of rice leaf sheath. mCherry fluorescence was observed using an Olympus FluoView FV1000-D confocal laser-scanning microscope (Olympus, Tokyo, Japan) equipped with a Multi argon laser, a HeNe G laser, a 40× UPlanSApo (0.9 numerical aperture) and a 60× UPlanFLN (0.9 numerical aperture) objective lens. To assess fluorescent signal in the reporter strains of *C. orbiculare*, conidia of the reporter strain were inoculated on the lower surfaces of cucumber cotyledons. Detection of mCherry fluorescence was performed using an Olympus FluoView FV500 confocal laser-scanning microscope (Olympus) with a Nikon 60× PlanApo (1.4 numerical aperture) oil-immersion objective (Nikon, Tokyo, Japan). Samples were mounted in water under cover slips and excited with the He/Ne laser. We used diachronic mirror DM488/543/633, SDM630 beam splitter, and emission filter BA560-600.

### Preparation of *M. oryzae*-infected rice leaf sheath extract and Western blot analysis

Conidial suspension (1×10^5^ conidia ml^−1^) was injected into rice (cv. Shin No. 2) leaf sheath and placed in a dew chamber at 25°C for 32 h in the dark. The infected leaf sheaths were ground in liquid nitrogen, thawed in X µl of extraction buffer (250 mM Tris-HCl pH 7.5, 2.5 mM EDTA, 0.1% ascorbic acid (w/v), 1 mM PMSF, 0.01% PI cocktail (v/v) (SIGMA-ALDRICH), 0.1% Triton X-100 (v/v)) for X mg sample, vortex for 10 min at 4°C, and centrifuged at 15,000× g for 20 min at 4°C in a microcentrifuge. The crude extracts (15 µl per lane) were separated on a 10–20% precast e-PAGEL (ATTO, Tokyo, Japan) and the proteins were transferred on to Immobilon Transfer Membranes (Millipore, Billerica, MA). The blots were blocked in 2% ECL Advance Blocking Agent (GE Healthcare, Buckinghamhire, UK) in TTBS (10 mM Tris-HCl, pH 7.5, 100 mM NaCl, 0.1% Tween 20 (v/v)) for 1 h at room temperature with gentle agitation. For immunodetection, blots were probed with anti-HA (3F10)-HRP (Roche, Mannheim, Germany) or anti-FLAG M2-HRP (SIGMA-ALDRICH) in a 1∶10,000 dillution in TTBS for 2 h. After washing the membrane for 10 min three times, the reactions were detected using an ECL Advance Western blotting detection reagents (GE Healthcare) and a Luminescent Image Analyzer LAS-4000 (Fujifilm, Tokyo, Japan).

### Preparation of culture filtrate and Western blot analysis

*M. oryzae* strains were cultured in 20 ml YG medium at 25°C at 120 rpm for 48 h. The culture was filtrated with Miracloth (Merck, Darmstadt, Germany), concentrated and desalted by ultrafiltration with Amicon Ultra-15 (10K) (Millipore). The culture filtrates (20 µg of protein per lane) were separated on a 12.5% SDS-PAGE gel and the proteins were transferred on to Immobilon Transfer Membranes (Millipore). The blots were blocked in 5% nonfat dry milk in TTBS for 1 h at room temperature with gentle agitation. For immunodetection, blots were probed with Living Colors DsRed Polyclonal Antibody (Clontech) in a 1∶1,000 dilution in TTBS for 2 h. After washing the membrane with TTBS for 10 min three times, Anti-Rabbit IgG HRP conjugate (Promega W401B) (Promega, Madison, WI) in a 1∶10,000 dilution in TTBS was used as secondary antibody and incubated for 1 h at room temperature with gentle agitation. After washing the membrane for 10 min three times, the reactions were detected using an ECL Western blotting detection reagents (GE Healthcare) and a Luminescent Image Analyzer LAS-4000 (Fujifilm).

### Accession numbers

Sequence data of MC69 and the homologs from this article can be found in the GenBank/EMBL data libraries accession number MGG_02848.6 (Mo), AB669186 (Co), EFQ29542 (Gg), EEY15898 (Va), EGY20943 (Vd), XP_965292 (Nc), EGO52621 (Nt), XP_003659994 (Mt), XP_00190740 (Pa), EFX05010 (Gc), EGU75378 (Fo), XP_388669 (Gz), EHK44387 (Ta), EHK23962 (Tv), EFY93067 (Mac), EFY97094 (Man), and EGX95034 (Cm).

## Supporting Information

Figure S1 Targeted gene disruption of *MC69*. (A) *MC69* locus and the disruption vector pGPSMC69-44. pGPSMC69-44 contains the GPS-HYG-CAM cassette (the hygromicin resistant gene HYG and the chloramphenicol resistant gene CAM) flanked by border sequences from *MC69*. (B) Genomic PCR analysis of wild-type Ina72 (lane 1, 10 and 19), three independent *mc69* mutants (lane 2∼4, 11∼13 and 20∼22), two independent *MC69* re-introduced strains (lane 5, 6, 14, 15, 23 and 24), wild-type 70-15 (lane 7, 16 and 25), two independent *mc69* mutants (lane 8, 9, 17, 18, 26 and 27). The transformants were analyzed by PCR with primers indicated in A (MC69F/MC69R, lane 1∼9), with HYG-specific primers (lane 10∼18) or with bialaphos-resistant gene specific primers (lane 19∼27).
(TIF)

Figure S2 Colony growth and conidiation of the *mc69* mutants. (A,C) Colony color and arial hyphae production of *mc69* mutants were normal. Photos were taken 7 days after incubation of wild type (Ina72), *mc69* mutants (*mc69-9*, *mc69-12* and *mc69-87*), wild type (70-15) and *mc69* mutants (*mc69-119* and *mc69-31*) on oatmeal agar. (B,D) Growth and conidiation of Ina72 (bar 1), *mc69-9* (bar 2), *mc69-12* (bar 3), *mc69-87* (bar 4), 70-15 (bar 5), *mc69-119* (bar 6) and *mc69-31* (bar 7). Mean values of colony diameter (cm) were measured 7 days of growth on oatmeal agar. Mean values are calculated from 3 replicates. Conidiogenesis was assessed in 3 replicate experiments. Means are expressed as numbers of conidia ×10^4^ of conidial suspension/cm^2^ of culture.
(TIF)

Figure S3 MC69 protein is produced in the invasive hyphae. (A,C) *In planta* growth of MC69::HA- and MC69::3xFLAG-expressing transformants (*mc69*+*MC69::HA* and *mc69*+*MC69::3xFLAG*) at the post-invasion stage. Invasive mycelia inside the rice (cv. Shin No. 2) leaf sheath cells were photographed 48 h after incubation. Scale bar = 20 µm. (B,D) Western blots probed with an anti-HA and an anti-FLAG antibodies. Protein extracts of rice leaf sheaths 24 h and 48 h after inoculation with Ina72 wild type (WT), *mc69*+*MC69::HA* and *mc69*+*MC69::3xFLAG* were loaded.
(TIF)

Figure S4 AVR-Pia avirulence function is retained under the *MC69* promoter. (A) The isolate Ina86-137 does not have AVR-Pia function and thus can cause disease on Sasanishiki harboring the *R* gene *Pia*. Ina86-137 strains transformed with *AVR-Piap::AVR-Pia* (+*AVR-Piap::AVR-Pia*) [20] or *MC69p::AVR-Pia* (+*MC69p::AVR-Pia-1*, *-2*) became imcompatible with Sasanishiki. Both Ina86-137 wild type, Ina86-137 containing *AVR-Piap::AVR-Pia*, or *MC69p::AVR-Pia* were able to cause disease on a rice cultivar Shin No. 2 lacking *Pia*, suggesting that the effect of transformation with *AVR-Piap::AVR-Pia* and *MC69p::AVR-Pia* is *Pia* dependent. (B) Confirmation of active *AVR-Pia* transgene by RT-PCR in *M. oryzae* transformants during infection. RT-PCR analysis of Ina86-137 WT (lane 1), +*AVR-Piap::AVR-Pia* (lane 2), +*MC69p::AVR-Pia-1* and *-2* (lane 3 and 4) with *AVR-Pia*- or *Mg-Actin*-specific primers [20].
(TIF)

Figure S5 MC69::mCherry confers BIC localization with weaker fluorescence than that of PWL2::mCherry. Merged DIC and mCherry images of rice leaf sheath cells infected by *M. oryzae* Sasa2 strain harboring (A) *PWL2p::PWL2::mCherry::NLS*, (B) *PWL2p::MC69::mCherry::NLS*, and (C) *PWL2p::MC69::mCherry* 27 h after inoculation as observed by confocal laser scanning microscopy. Arrows indicate BICs and triangles indicate rice nuclei. Pinhole settings are 80 µm for left panels and 240 µm for right panels. Scale bar = 20 µm.
(TIF)

Figure S6 Predicted amino acid sequence alignment of MC69 with homologs from other filamentous fungi. Amino acid sequences of MC69 (Mo), MC69 homologs of *Colletotrichum orbiculare* (Co), *Glomerella graminicola* (Gg), *Verticillium albo-atrum* (Va), *V. dahliae* (Vd), *Neurospora crassa* (Nc), *N. tetrasperma* (Nt), *Myceliophthora thermophila* (Mt), *Podospora anserina* (Pa), *Grosmannia clavigera* (Gc), *Fusarium oxysporum* (Fo), *Gibberella zeae* (Gz), *Trichoderma atroviride* (Ta), *T. virens* (Tv), *Metarhizium acridum* (Mac), *M. anisopliae* (Man) and *Cordyceps militaris* (Cm) were aligned using the Clustal W program [52]. Identical amino acids are indicated as white letters on a black background. Similar residues are shown on gray backgrounds. Gaps introduced for alignment are indicated by dashes. The predicted signal peptide and two conserved cysteine residues (C36 and C46) are indicated on top.
(TIF)

Figure S7 Phylogenetic tree of *M. oryzae* MC69 protein sequence and 16 homologs from other fungi. Phylogenetic analyses were performed with *M. oryzae* MC69 (Mo), with 16 homologs are shown in Figure S6 legend.
(TIF)

Figure S8 Invasive growth rating of rice leaf sheath cells 32 h after inoculating with Ina72 WT, *mc69*, *mc69*+*MC69*, *mc69*+*MC69(C36A)*, *mc69*+*MC69(C46A)* and *mc69*+*MC69(C36A,C46A)*. For details of the invasive growth levels and rating see Materials and Methods.
(TIF)

Figure S9 Invasive growth rating of rice leaf sheath cells 32 h after inoculating with 70-15 WT and *mc69-31*. For details of the invasive growth levels and rating see Materials and Methods.
(TIF)

Figure S10 Intron/exon organization in *M. oryzae MC69* gene and 16 orthologous genes from other fungi. Abbreviations of fungus names are shown in Figure S6 legend.
(TIF)

Figure S11 Gene disruption of *CoMC69* in *C. orbiculare*. (A) *CoMC69* locus and the gene disruption vector pGDCOMC69. By homologous recombination through double crossing over, the *CoMC69* gene was replaced by a hygromycin resistance gene cassette (HYG). (B) Genomic PCR analysis of the *Comc69* mutants of *C. orbiculare*. Genomic DNAs were isolated from the wild-type strain 104-T and the *comc69* strains (DMC1 and DMC2). The 0.3 kb product containing the entire *CoMC69* gene was amplified from the genome DNA of 104-T with the two primers, indicated by arrows, COMC69F (5′-CGAAAGCAAGGCAGCTATTC-3′) and COMC69R (5′-CTCAGAGGACTACAGACATG-3′). In contrast, the 1.6 kb product was amplified from the genome DNA of both *comc69* strains, which is consistent with gene replacement shown in (A). Lane 1, λ *Hin*d III marker; lane 2, 104-T; lane 3, DMC1; lane 4, DMC2. (C) Colony phenotype of the *C. orbiculare mc69* mutants. The wild-type strain 104-T and *Comc69* mutants (DMC1 and DMC2) were grown on PDA for 12 days.
(TIF)

Table S1 SuperSAGE result of cAMP-treated *Magnaporthe oryzae* strain 70-15.
(XLS)
