# Supplementary figures and images for: Large-Scale Gene Disruption in Magnaporthe oryzae Identifies MC69, a Secreted Protein Required for Infection by Monocot and Dicot Fungal Pathogens
Source: PLoS Pathog. 2012 May 10;8(5):e1002711. doi: 10.1371/journal.ppat.1002711 (PMC3349759; doi:10.1371/journal.ppat.1002711)

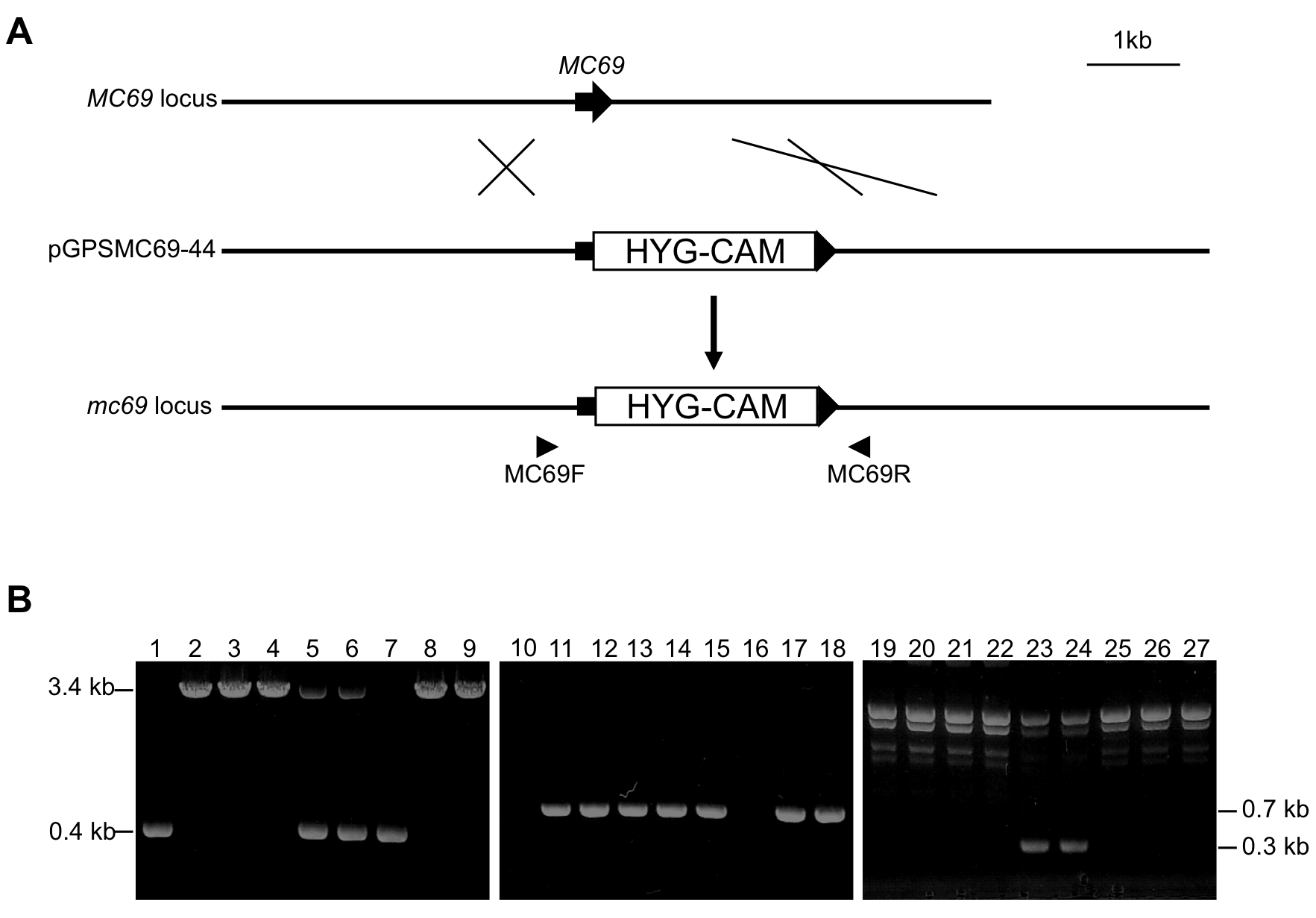

Supplement: Figure S1 — Targeted gene disruption of MC69. (A) MC69 locus and the disruption vector pGPSMC69-44. pGPSMC69-44 contains the GPS-HYG-CAM cassette (the hygromicin resistant gene HYG and the chloramphenicol resistant gene CAM) flanked by border sequences from MC69. (B) Genomic PCR analysis of wild-type Ina72 (lane 1, 10 and 19), three independent mc69 mutants (lane 2∼4, 11∼13 and 20∼22), two independent MC69 re-introduced strains (lane 5, 6, 14, 15, 23 and 24), wild-type 70-15 (lane 7, 16 and 25), two independent mc69 mutants (lane 8, 9, 17, 18, 26 and 27). The transformants were analyzed by PCR with primers indicated in A (MC69F/MC69R, lane 1∼9), with HYG-specific primers (lane 10∼18) or with bialaphos-resistant gene specific primers (lane 19∼27). (TIF) [file ppat.1002711.s001.tif]

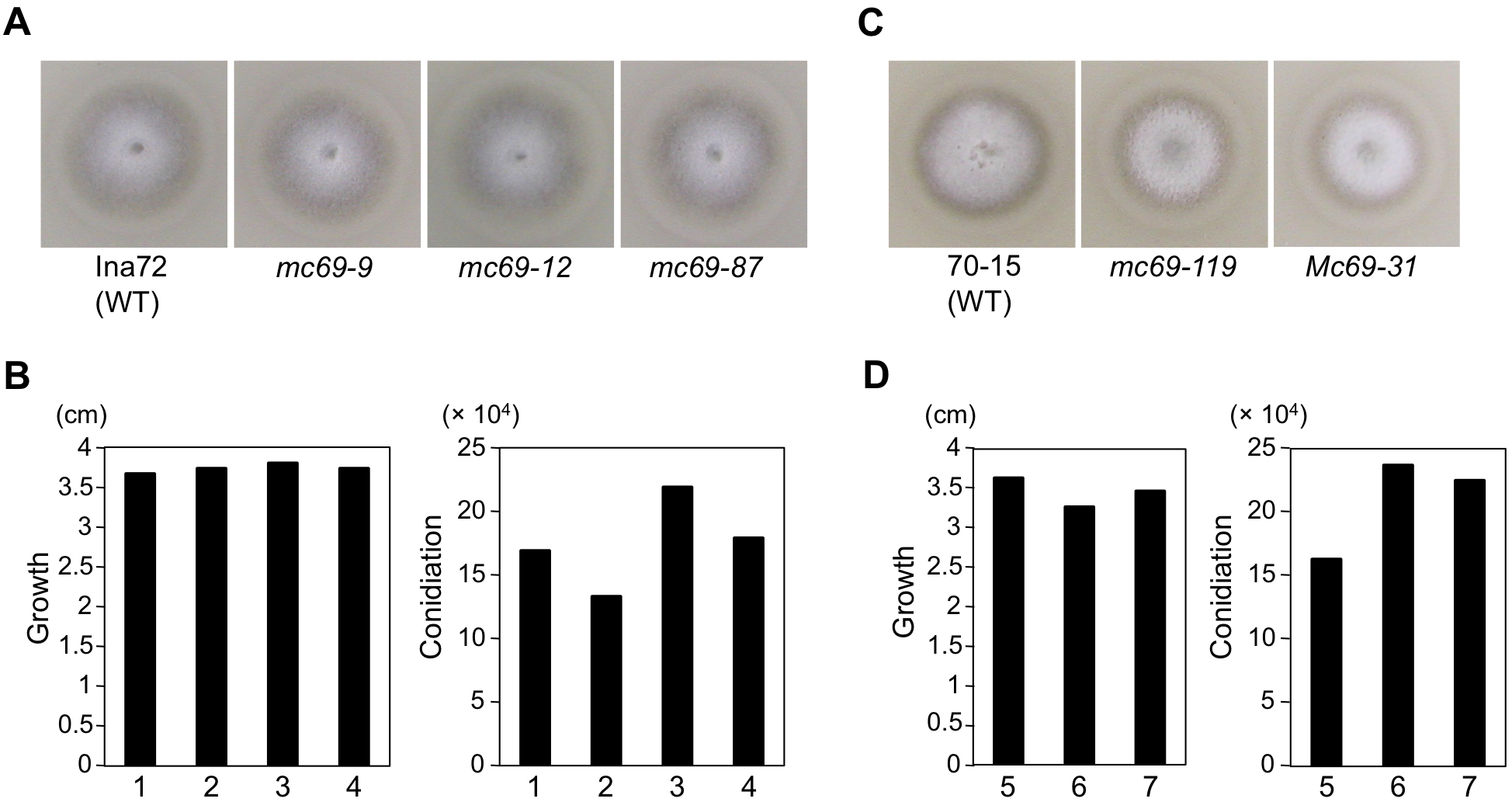

Supplement: Figure S2 — Colony growth and conidiation of the mc69 mutants. (A,C) Colony color and arial hyphae production of mc69 mutants were normal. Photos were taken 7 days after incubation of wild type (Ina72), mc69 mutants (mc69-9, mc69-12 and mc69-87), wild type (70-15) and mc69 mutants (mc69-119 and mc69-31) on oatmeal agar. (B,D) Growth and conidiation of Ina72 (bar 1), mc69-9 (bar 2), mc69-12 (bar 3), mc69-87 (bar 4), 70-15 (bar 5), mc69-119 (bar 6) and mc69-31 (bar 7). Mean values of colony diameter (cm) were measured 7 days of growth on oatmeal agar. Mean values are calculated from 3 replicates. Conidiogenesis was assessed in 3 replicate experiments. Means are expressed as numbers of conidia ×104 of conidial suspension/cm2 of culture. (TIF) [file ppat.1002711.s002.tif]

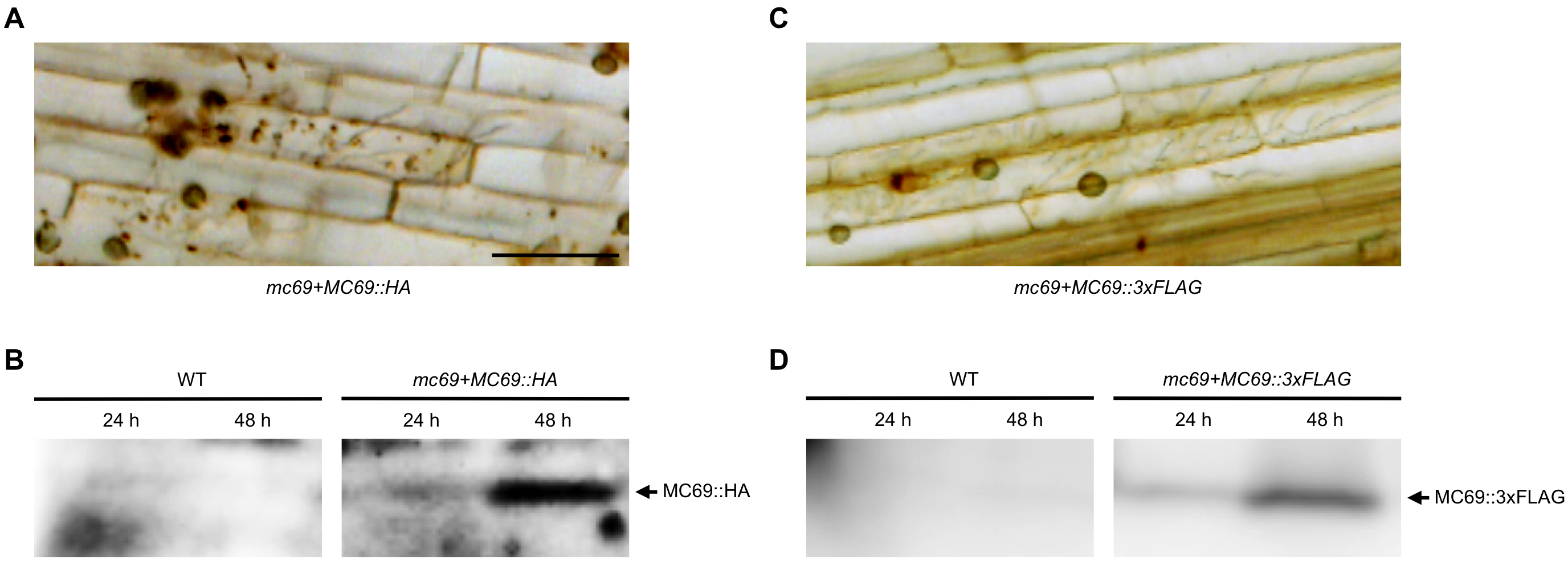

Supplement: Figure S3 — MC69 protein is produced in the invasive hyphae. (A,C) In planta growth of MC69::HA- and MC69::3xFLAG-expressing transformants (mc69+MC69::HA and mc69+MC69::3xFLAG) at the post-invasion stage. Invasive mycelia inside the rice (cv. Shin No. 2) leaf sheath cells were photographed 48 h after incubation. Scale bar = 20 µm. (B,D) Western blots probed with an anti-HA and an anti-FLAG antibodies. Protein extracts of rice leaf sheaths 24 h and 48 h after inoculation with Ina72 wild type (WT), mc69+MC69::HA and mc69+MC69::3xFLAG were loaded. (TIF) [file ppat.1002711.s003.tif]

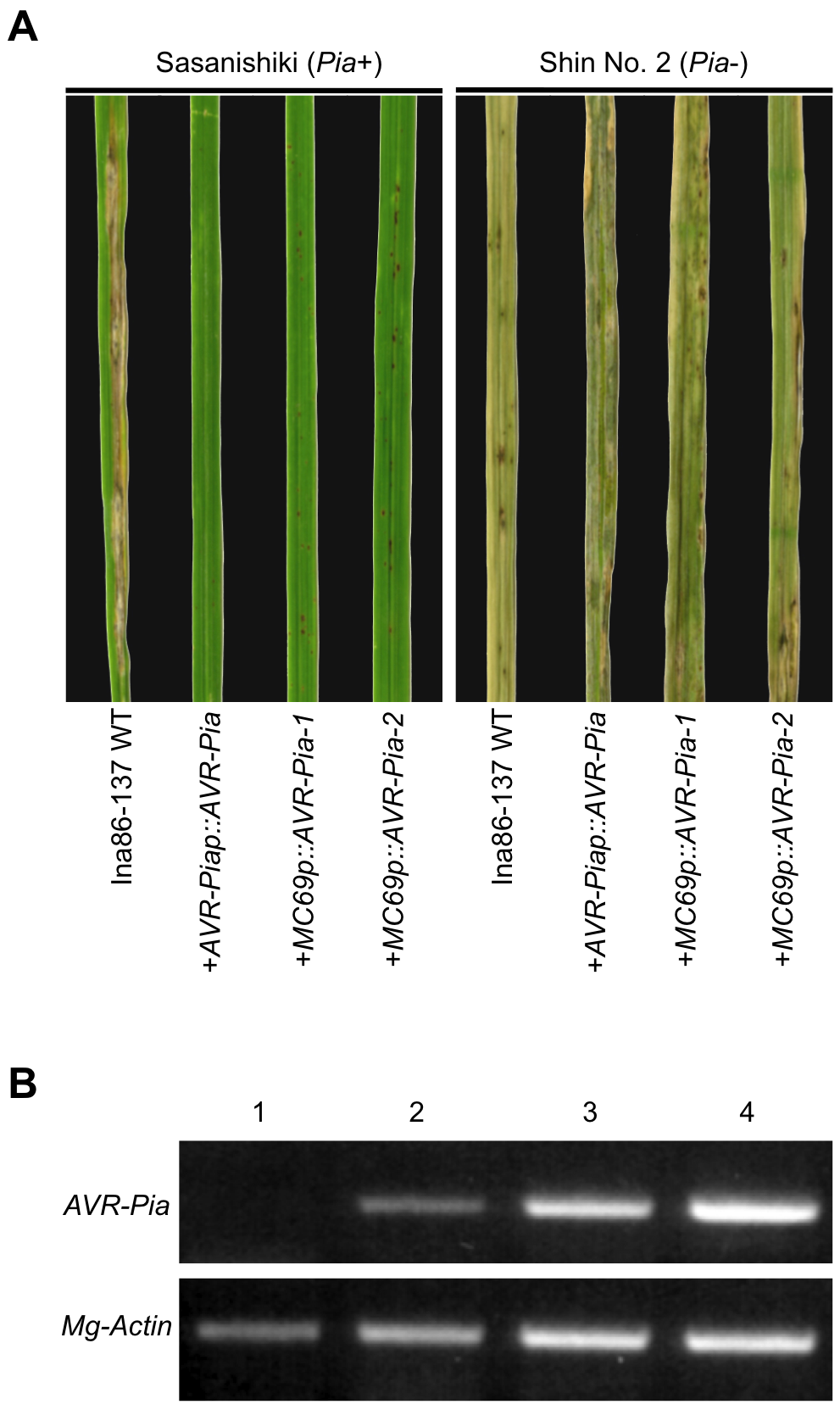

Supplement: Figure S4 — AVR-Pia avirulence function is retained under the MC69 promoter. (A) The isolate Ina86-137 does not have AVR-Pia function and thus can cause disease on Sasanishiki harboring the R gene Pia. Ina86-137 strains transformed with AVR-Piap::AVR-Pia (+AVR-Piap::AVR-Pia) [20] or MC69p::AVR-Pia (+MC69p::AVR-Pia-1, -2) became imcompatible with Sasanishiki. Both Ina86-137 wild type, Ina86-137 containing AVR-Piap::AVR-Pia, or MC69p::AVR-Pia were able to cause disease on a rice cultivar Shin No. 2 lacking Pia, suggesting that the effect of transformation with AVR-Piap::AVR-Pia and MC69p::AVR-Pia is Pia dependent. (B) Confirmation of active AVR-Pia transgene by RT-PCR in M. oryzae transformants during infection. RT-PCR analysis of Ina86-137 WT (lane 1), +AVR-Piap::AVR-Pia (lane 2), +MC69p::AVR-Pia-1 and -2 (lane 3 and 4) with AVR-Pia- or Mg-Actin-specific primers [20]. (TIF) [file ppat.1002711.s004.tif]

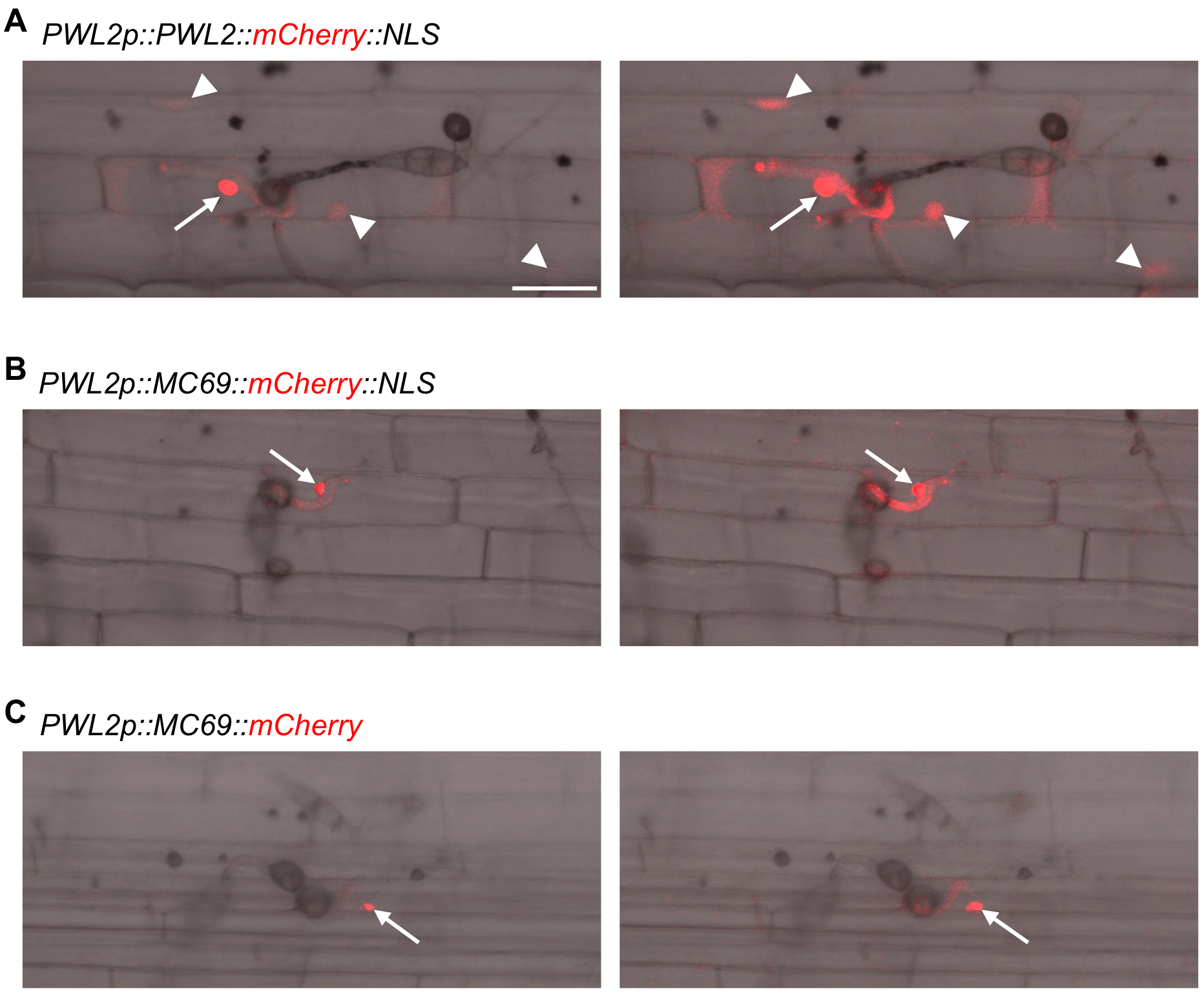

Supplement: Figure S5 — MC69::mCherry confers BIC localization with weaker fluorescence than that of PWL2::mCherry. Merged DIC and mCherry images of rice leaf sheath cells infected by M. oryzae Sasa2 strain harboring (A) PWL2p::PWL2::mCherry::NLS, (B) PWL2p::MC69::mCherry::NLS, and (C) PWL2p::MC69::mCherry 27 h after inoculation as observed by confocal laser scanning microscopy. Arrows indicate BICs and triangles indicate rice nuclei. Pinhole settings are 80 µm for left panels and 240 µm for right panels. Scale bar = 20 µm. (TIF) [file ppat.1002711.s005.tif]

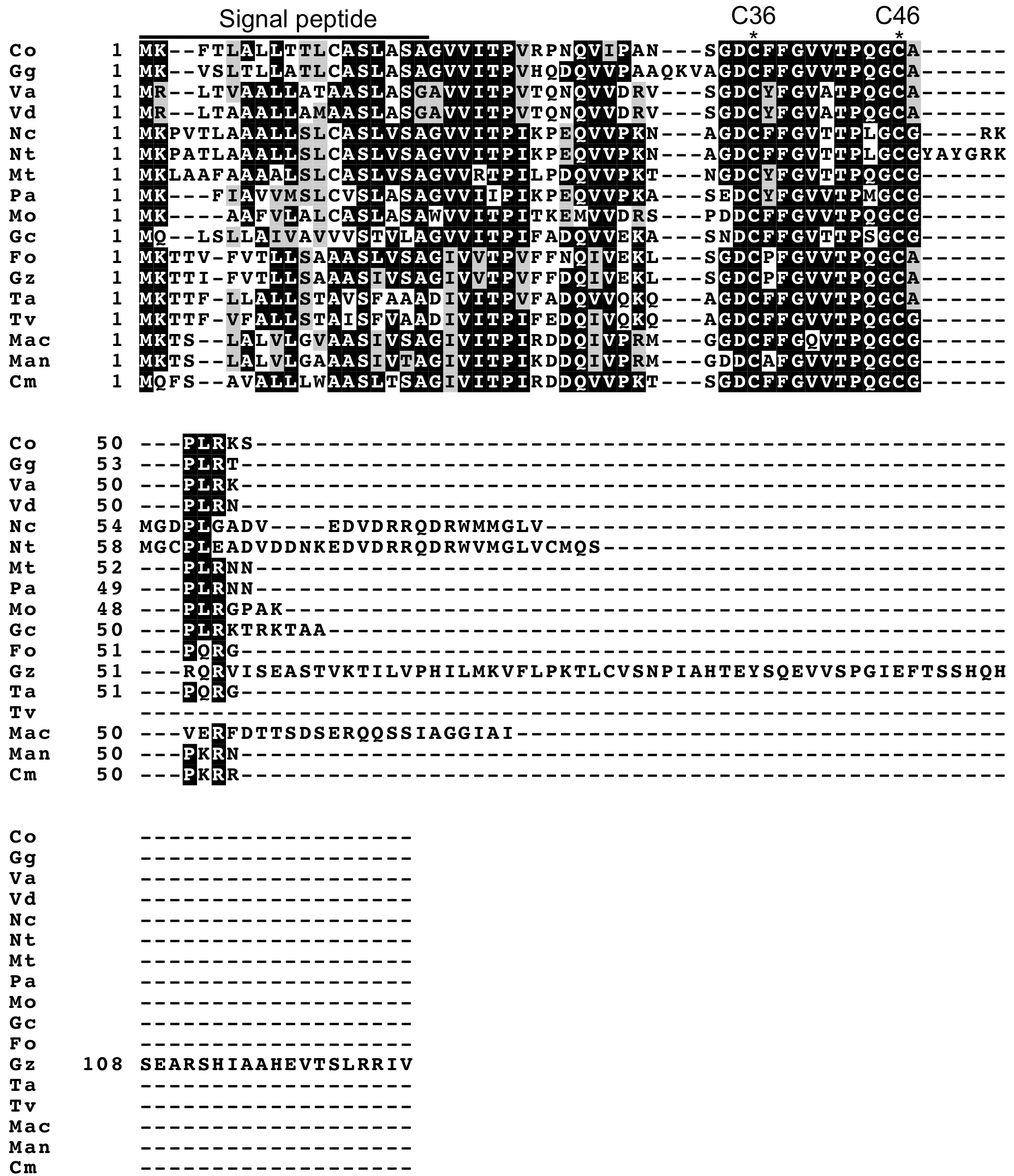

Supplement: Figure S6 — Predicted amino acid sequence alignment of MC69 with homologs from other filamentous fungi. Amino acid sequences of MC69 (Mo), MC69 homologs of Colletotrichum orbiculare (Co), Glomerella graminicola (Gg), Verticillium albo-atrum (Va), V. dahliae (Vd), Neurospora crassa (Nc), N. tetrasperma (Nt), Myceliophthora thermophila (Mt), Podospora anserina (Pa), Grosmannia clavigera (Gc), Fusarium oxysporum (Fo), Gibberella zeae (Gz), Trichoderma atroviride (Ta), T. virens (Tv), Metarhizium acridum (Mac), M. anisopliae (Man) and Cordyceps militaris (Cm) were aligned using the Clustal W program [52]. Identical amino acids are indicated as white letters on a black background. Similar residues are shown on gray backgrounds. Gaps introduced for alignment are indicated by dashes. The predicted signal peptide and two conserved cysteine residues (C36 and C46) are indicated on top. (TIF) [file ppat.1002711.s006.tif]

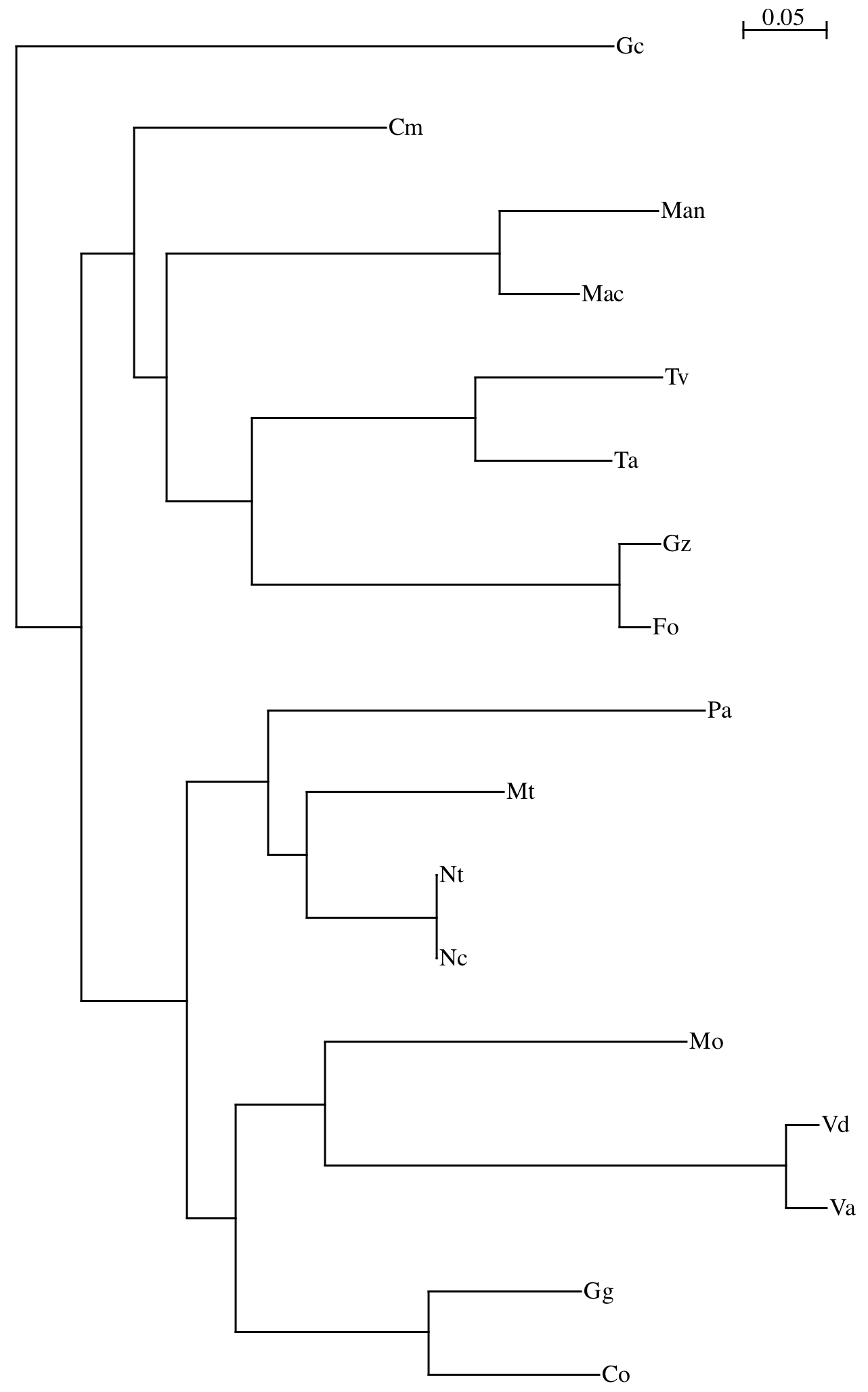

Supplement: Figure S7 — Phylogenetic tree of M. oryzae MC69 protein sequence and 16 homologs from other fungi. Phylogenetic analyses were performed with M. oryzae MC69 (Mo), with 16 homologs are shown in Figure S6 legend. (TIF) [file ppat.1002711.s007.tif]

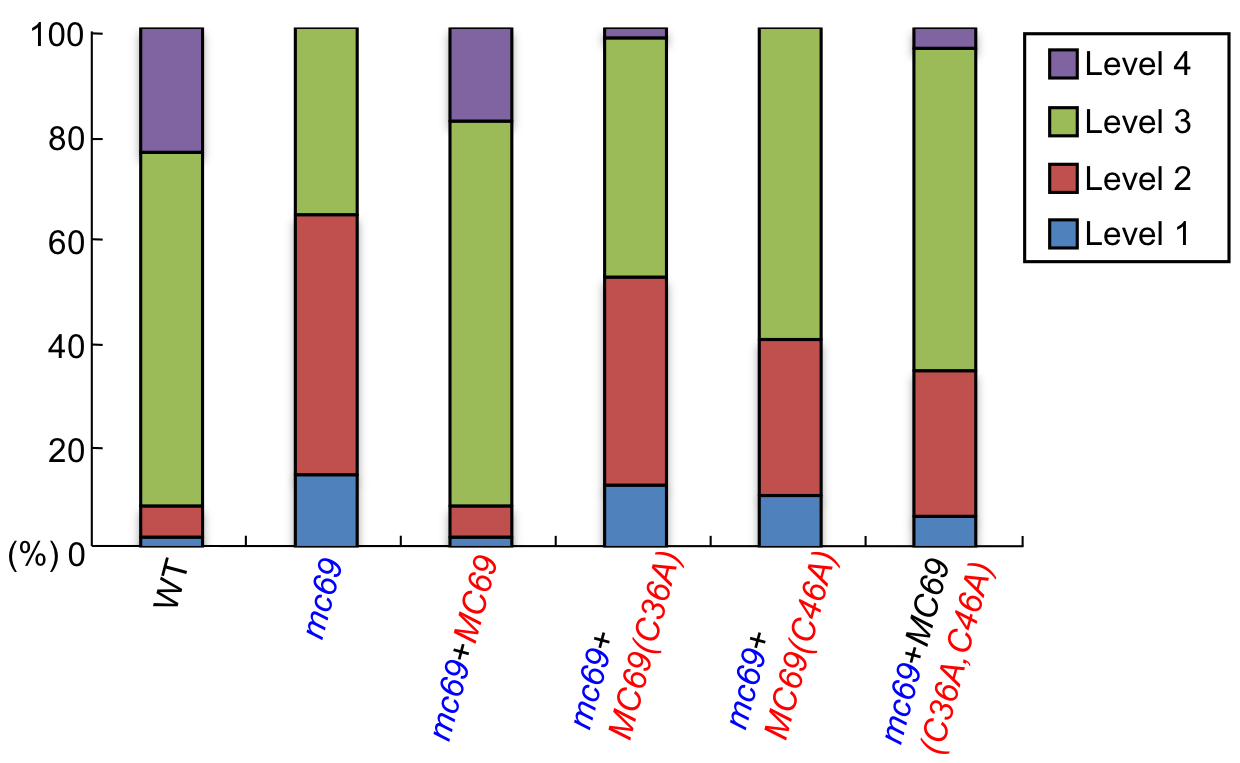

Supplement: Figure S8 — Invasive growth rating of rice leaf sheath cells 32 h after inoculating with Ina72 WT, mc69, mc69+MC69, mc69+MC69(C36A), mc69+MC69(C46A) and mc69+MC69(C36A,C46A). For details of the invasive growth levels and rating see Materials and Methods. (TIF) [file ppat.1002711.s008.tif]

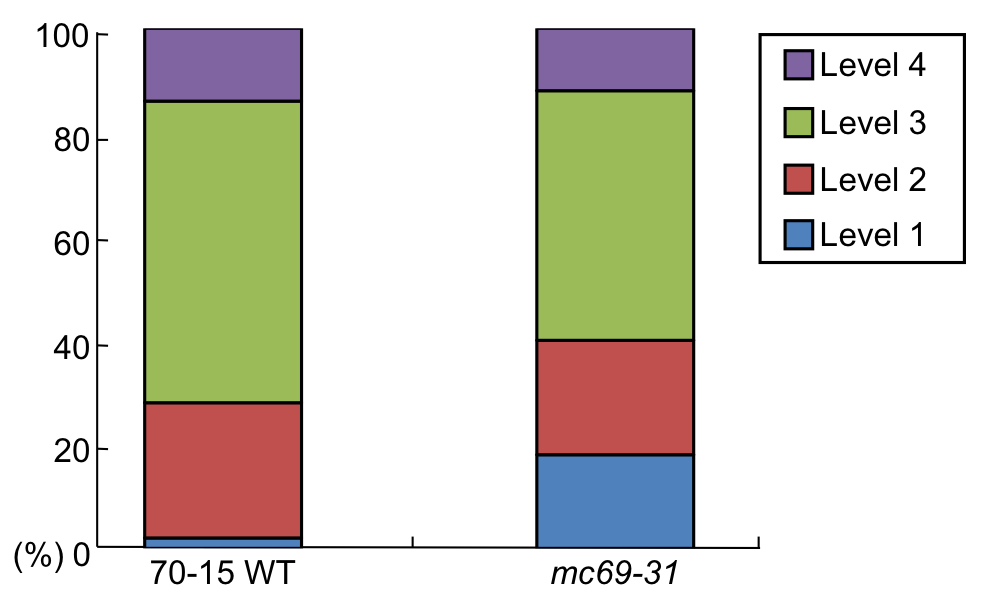

Supplement: Figure S9 — Invasive growth rating of rice leaf sheath cells 32 h after inoculating with 70-15 WT and mc69-31. For details of the invasive growth levels and rating see Materials and Methods. (TIF) [file ppat.1002711.s009.tif]

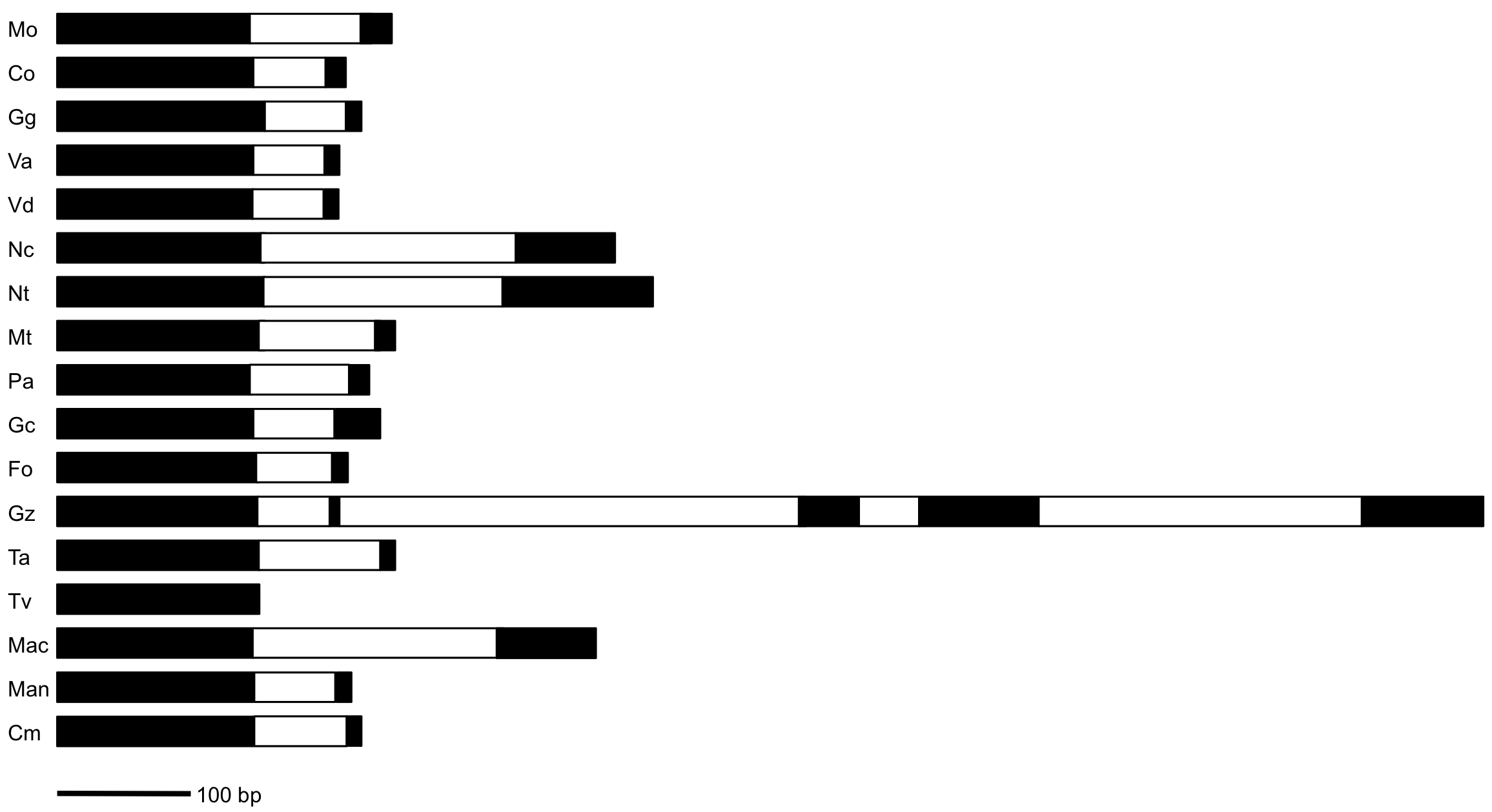

Supplement: Figure S10 — Intron/exon organization in M. oryzae MC69 gene and 16 orthologous genes from other fungi. Abbreviations of fungus names are shown in Figure S6 legend. (TIF) [file ppat.1002711.s010.tif]

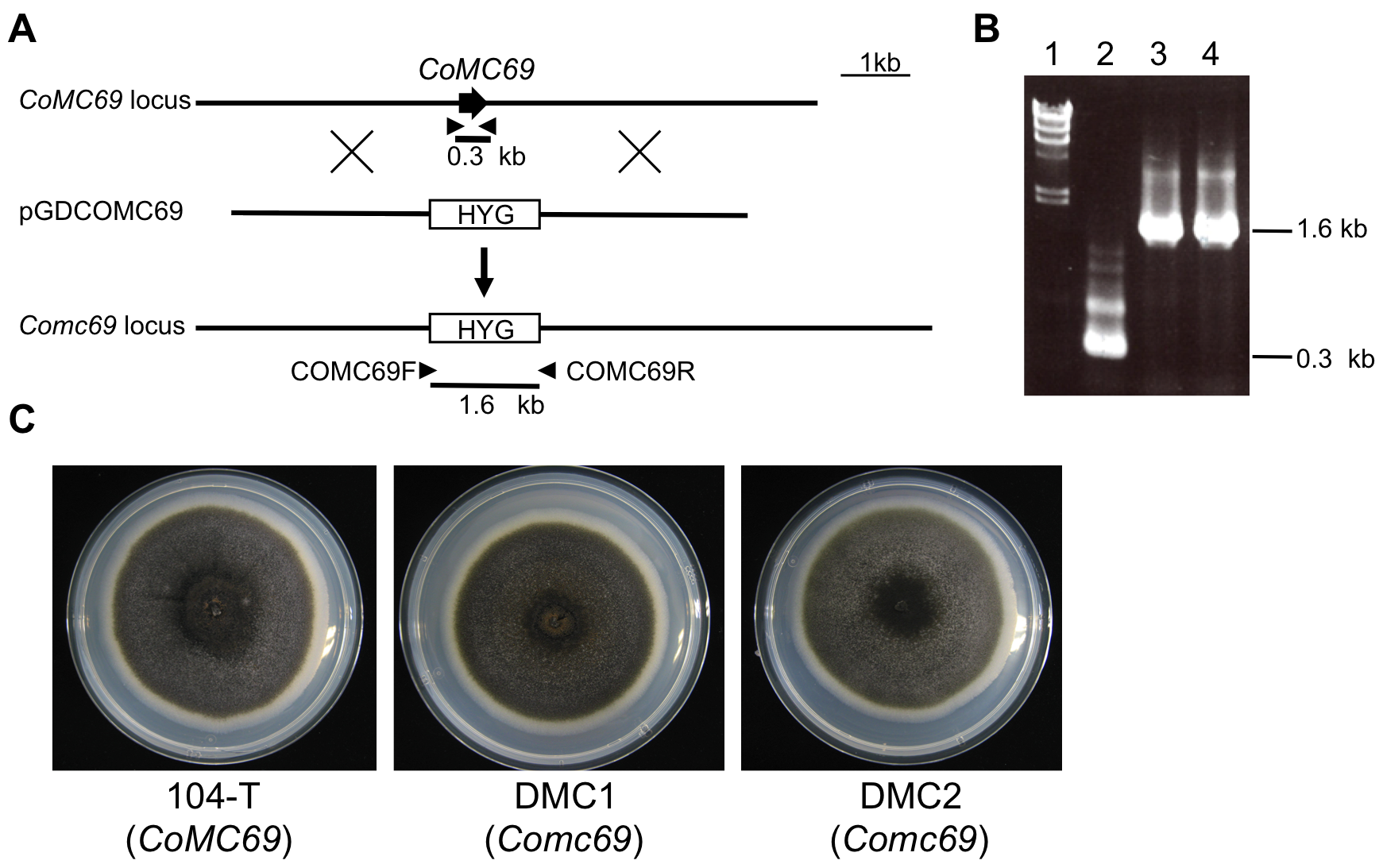

Supplement: Figure S11 — Gene disruption of CoMC69 in C. orbiculare. (A) CoMC69 locus and the gene disruption vector pGDCOMC69. By homologous recombination through double crossing over, the CoMC69 gene was replaced by a hygromycin resistance gene cassette (HYG). (B) Genomic PCR analysis of the Comc69 mutants of C. orbiculare. Genomic DNAs were isolated from the wild-type strain 104-T and the comc69 strains (DMC1 and DMC2). The 0.3 kb product containing the entire CoMC69 gene was amplified from the genome DNA of 104-T with the two primers, indicated by arrows, COMC69F (5′-CGAAAGCAAGGCAGCTATTC-3′) and COMC69R (5′-CTCAGAGGACTACAGACATG-3′). In contrast, the 1.6 kb product was amplified from the genome DNA of both comc69 strains, which is consistent with gene replacement shown in (A). Lane 1, λ Hind III marker; lane 2, 104-T; lane 3, DMC1; lane 4, DMC2. (C) Colony phenotype of the C. orbiculare mc69 mutants. The wild-type strain 104-T and Comc69 mutants (DMC1 and DMC2) were grown on PDA for 12 days. (TIF) [file ppat.1002711.s011.tif]
